# Deep Generative Modeling for Cognitive Diagnosis via Exploratory DeepCDMs

**DOI:** 10.1017/psy.2025.10065

**Published:** 2025-12-17

**Authors:** Jia Liu, Yuqi Gu

**Affiliations:** Department of Statistics, Columbia University, USA

**Keywords:** deep cognitive diagnostic models (DeepCDMs), deep generative modeling, exploratory cognitive diagnosis, identifiability, layer-wise EM algorithm

## Abstract

Deep generative modeling is a powerful framework in modern machine learning, renowned for its ability to use latent representations to predict and generate complex high-dimensional data. Its advantages have also been recognized in psychometrics. In this article, we substantially extend the deep cognitive diagnostic models (DeepCDMs) in Gu (*Psychometrika*, 89:118–150, 2024) to challenging exploratory scenarios with deeper structures and all 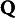 -matrices unknown. The exploratory DeepCDMs can be viewed as an adaptation of deep generative models (DGMs) toward diagnostic purposes. Compared to classic DGMs, exploratory DeepCDMs enjoy critical advantages, including identifiability, interpretability, parsimony, and sparsity, which are all necessary for diagnostic modeling. We propose a novel layer-wise expectation–maximization (EM) algorithm for parameter estimation, incorporating layer-wise nonlinear spectral initialization and 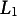 penalty terms to promote sparsity. From a parameter estimation standpoint, this algorithm reduces sensitivity to initial values and mitigates estimation bias that commonly affects classical approaches for deep latent variable models. Meanwhile, from an algorithmic perspective, our method presents an original layer-wise EM framework, inspired by modular training in DGMs but uniquely designed for the structural and interpretability demands of diagnostic modeling. Extensive simulation studies and real data applications illustrate the effectiveness and efficiency of the proposed method.

## 1 Introduction

Over the past two decades, cognitive diagnosis models (CDMs) have become increasingly prominent in educational and psychological measurement (e.g., Chen et al., 2015; de la Torre, 2011; Henson et al., 2009; Junker & Sijtsma, 2001; Rupp et al., 2010; von Davier, 2008; von Davier & Lee, 2019). CDMs are a class of psychometric models that use item response data to infer examinees’ mastery status on multiple discrete latent *attributes*, such as skills, subskills, or diagnostic criteria. In most applications, each attribute is assumed to be binary, representing the presence or absence of a specific cognitive ability or psychological trait. By estimating an individual’s profile across these attributes, CDMs facilitate detailed diagnostic reporting. This information enables practitioners and educators to identify students’ strengths and weaknesses at a granular level, supporting the design of targeted interventions and more individualized feedback.

Recently, interest in adopting higher-order structures for CDMs has grown, aiming to capture interdependencies between the latent attributes (de la Torre & Douglas, 2004; de la Torre & Song, 2009; Templin et al., 2008). Most existing models adopt a single layer of higher-order continuous latent traits to explain correlations among lower-level latent attributes (e.g., Bradshaw & Templin, 2014; de la Torre & Douglas, 2004; Liu et al., 2025; Ma, 2022; Templin et al., 2008). Although these single-layer higher-order models offer an interpretable and simplified representation of attribute dependencies, they may be limited in modeling deeper latent hierarchies or providing more granular cognitive diagnoses. To model deeper-level cognitive processes, the recent deep cognitive diagnostic models (DeepCDMs) proposed by Gu (2024) employ a deep architecture to capture probabilistic relationships across *multiple discrete latent* layers. DeepCDMs flexibly let each of these layers deliver diagnostic information at a distinct level of granularity. Despite this added depth, DeepCDMs remain parsimonious through compact parameterization and are mathematically identifiable under intuitive conditions.

In this article, we show that the advantages of DeepCDMs can be further realized by generalizing them to a challenging exploratory setting, where the attribute relationships between adjacent layers (i.e., all the 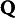 -matrices) are unknown. The exploratory DeepCDMs can be viewed as an adaptation of deep generative models (DGMs) for psychometrics and educational measurement, with identifiability constraints imposed to serve diagnostic purposes. DeepCDMs share structural similarities with several existing DGMs, such as deep belief networks (DBNs; Hinton et al., 2006) (see Section 2.3 for further discussion). This connection highlights the expressive power of DeepCDMs from the perspective of DGMs. In particular, DeepCDM’s layered architecture defines a hierarchical generative process, suitable for modeling students’ hierarchical and heterogeneous cognitive processes behind data. This structure enables DeepCDMs to approximate highly complex response distributions while maintaining a tractable form for layer-wise learning.

Although usual DGMs (Hinton et al., 2006; Salakhutdinov & Hinton, 2009) excel at predictive and generative performance, their architectures and estimation algorithms are often heuristically designed and lack rigorous statistical foundations. Importantly, whether the parameters underlying the latent representations are uniquely identifiable is largely unknown for DGMs. This gap motivates us to introduce exploratory DeepCDMs, which are built for diagnostic purposes and are *fully identifiable*. Exploratory DeepCDMs are identifiable under transparent conditions on the between-layer 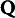 -matrices (Gu, 2024). Identifiability ensures that no two distinct parameter sets yield the same marginal distribution of the observed responses, thereby guaranteeing consistent parameter estimation. As a consequence, DeepCDMs can provide statistically reliable personalized diagnoses of hierarchical latent abilities. The identifiability conditions naturally imply an interpretable shrinking-ladder-shaped sparse deep architecture, enabling the model to capture the latent skills from fine-grained (shallower and closer to the response data layer) to coarse-grained (deeper and more higher-order). Statistically, such architectures also induce parsimonious parameterizations, crucial for reflecting test design constraints in real-world educational assessments.

Parameter estimation is a challenging issue for exploratory DeepCDMs, as the parameters and 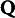 -matrices across all layers need to be estimated. The commonly used estimation methods for related hierarchical models are Markov chain Monte Carlo (MCMC; Robert and Casella, 2004) method and expectation–maximization (EM) algorithm (Dempster et al., 1977). Gu (2024) employed MCMC to estimate confirmatory DeepCDMs with known 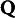 -matrices. For exploratory DeepCDMs, MCMC can, in principle, be developed by incorporating additional sampling steps for the 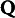 -matrix entries. However, when the 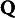 -matrices are unknown and the latent structure involves more than two layers—as in the settings considered in this work—significant practical challenges, such as initialization sensitivity, slower convergence, MCMC mixing difficulties, and increased computational cost, may limit its scalability and efficiency. The classical EM, as explained later in Section 3.4, though faster, suffers from (a) extreme sensitivity to initialization, since all parameters must be initialized simultaneously in a highly nonconvex, multi-layer parameter space and (b) cyclic bias accumulation, where errors in one layer’s estimation propagate through both the E- and M-steps into other layers over successive iterations. On the other hand, although many algorithms have been proposed for general DGMs in machine learning (e.g., Hinton et al., 2006; Le Roux & Bengio, 2008; Ranganath et al., 2015; Salakhutdinov & Hinton, 2009), they are not directly applicable to DeepCDMs, as their typically overparameterized architectures do not satisfy the parsimony and identifiability requirements of diagnostic modeling and are not designed to promote sparsity or interpretability.

In this work, we propose a novel layer-wise EM algorithm for regularized maximum likelihood estimation with a layerwise 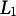 penalty in exploratory DeepCDMs. The algorithm estimates parameters and 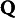 -matrices sequentially, starting from the bottom layer, where a one-layer EM algorithm is used to estimate both the between-layer coefficients and the proportion parameters of the latent attributes. These proportion parameters are then used to generate pseudo-samples of latent attributes, which serve as input for the next layer. We will continue this process one layer after another until all layers are estimated. This strategy is not only intuitive but also grounded in the model’s generative structure: marginalizing out deeper layers naturally yields a standard one-layer CDM at the bottom, justifying the use of a one-layer EM for its estimation. In deeper layers, each step builds on the most informative signals from the previous one—either as estimated distributions or generated pseudo-observations—thus respecting the model’s hierarchical nature. Interestingly, the identifiability proof shows that identifiability can be examined and established in a layer-by-layer manner, thanks to the formulation of the directed graphical model and the discrete nature of the latent attributes. This theoretical insight also supports the design of our proposed algorithm and provides a solid foundation for treating imputed attributes as if observed in each step. Additionally, our layerwise estimation strategy conceptually aligns with the modular training principles widely used in deep generative modeling, where complex models are progressively trained through simpler, localized components. A more detailed discussion on this is in Section 3.5.2.

Initialization plays a crucial role in EM-based estimation, particularly in exploratory settings where the 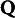 -matrices are unknown and must be estimated. In such cases, the parameter space becomes more complex, and a well-informed initialization can greatly enhance convergence stability and estimation quality. To this end, we adopt a fast, non-iterative procedure based on universal singular value thresholding (USVT), which yields reliable starting values with theoretical guarantees under certain conditions (Chatterjee, 2015; Zhang et al., 2020). The initialization is conducted in a sequential, layer-by-layer manner. For each layer, the input matrix is first denoised via truncated SVD, then linearized by applying the inverse link function, and then a second SVD followed by Varimax rotation is applied to recover a sparse coefficient matrix, promoting sparsity and identifiability. We adopt a penalized estimation framework where all 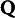 -matrices are treated as unknown and estimated from data. At each layer, 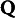 -matrix estimation is framed as a latent variable selection problem, with an 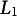 penalty imposed on the coefficient parameters to encourage sparsity. The M-step of each layer’s EM update is solved via cyclical coordinate descent (Friedman et al., 2010; Tay et al., 2023), efficiently maximizing the penalized log-likelihood. Additionally, as discussed in Section 3.6, although the algorithm is developed under an exploratory framework, it can be readily adapted for confirmatory applications. Our extensive simulation studies demonstrate the excellent performance of the proposed layer-wise EM in challenging scenarios involving three latent layers. Finally, we illustrate the practical utility of exploratory DeepCDM using data from the 2019 Trends in International Mathematics and Science Study (TIMSS) assessment.

The remainder of this article is organized as follows. Section 2 introduces the exploratory DeepCDMs framework, discusses its formulation as a DGM, and addresses the identifiability issues. Section 3 presents an efficient layer-wise algorithm for parameter estimation. Section 4 presents simulation studies to evaluate the performance of the proposed layer-wise EM algorithm for exploratory DeepCDMs under various measurement models. Section 5 applies the proposed method to real data from the TIMSS 2019 assessment. Finally, Section 6 gives concluding remarks.

## 2 Exploratory DeepCDMs framework

In this section, we present the exploratory DeepCDM framework. We will build on the concepts of confirmatory DeepCDMs in Gu (2024) and provide additional details for the exploratory setting. We then discuss how exploratory DeepCDMs adapt DGMs for psychometrics, highlighting their architectural similarities and the additional structural constraints for facilitating diagnostic feedback. Finally, we discuss the theoretical identifiability of the model, with formal results provided in the Supplementary Material.

### 2.1 Model setup

The DeepCDM framework is developed to address the need for diagnostic modeling at multiple granularities. It is defined using the terminology of probabilistic graphical models (Koller & Friedman, 2009; Wainwright & Jordan, 2008), particularly directed graphical models. These models employ graphs to compactly represent the joint distribution of high-dimensional random variables, where nodes correspond to variables and edges encode their direct probabilistic relationships.

We first review the definition of a *directed acyclic graph* (DAG), also referred to as a Bayesian network (Pearl, 1988). In a DAG, every edge has a direction, and no directed cycles are allowed. Consider *M* random variables, 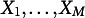 , which correspond to *M* nodes in the graph. If a directed edge goes from 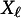 to 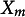 , we say that 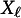 is a *parent* of 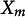 , and 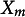 is a *child* of 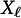 . Let 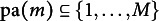 denote the index set of all parents of 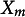 . Define 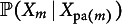 as the conditional distribution of 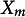 given its parents 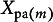 . Based on this DAG structure, the joint distribution of 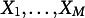 factorizes as follows: 
(1)

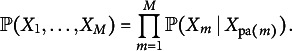



**Figure 1:**
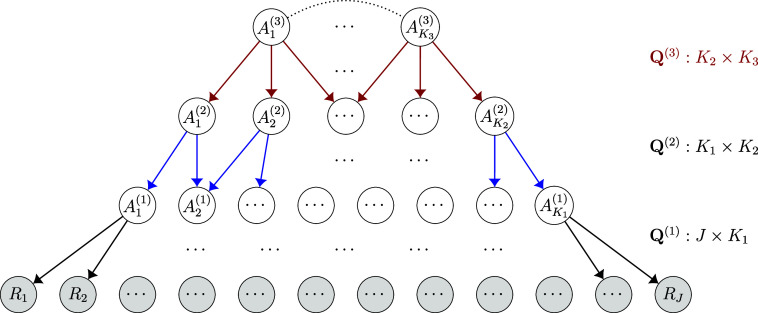
A ladder-shaped three-latent-layer DeepCDM. *Note*: Gray nodes are observed variables, and white nodes are latent ones. Multiple layers of binary latent variables 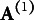 , 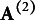 , and 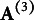 successively generate the observed binary responses 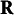 . Binary matrices 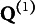 , 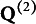 , and 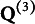 encode the sparse connection patterns between adjacent layers in the graph.

We now present the general DeepCDM formulation. For a DeepCDM with *D* latent layers, we denote the *d*-th latent layer as 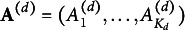 for each 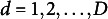 , where larger *d* correspond to deeper layers. In DeepCDMs, all edges are directed top-down and occur only between adjacent layers, defining a generative process from high-level latent variables to observed responses. Specifically, the bottom layer consists of the observed response variables for the *J* items, denoted as 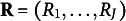 . The first latent layer, right above the bottom layer, captures the most fine-grained latent attributes, represented as 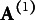 . These are generated from the second latent layer 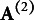 , and the process continues recursively up to the deepest layer 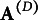 . Figure 1 gives an example of a DeepCDM with three latent layers ( 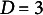 ). Given the variables in the layer right above, the variables within each layer of the DeepCDM are conditionally independent. This structure intuitively models how more specific latent skills are successively derived from more general, higher-level latent “meta-skills.” A natural assumption, supported by the model’s identifiability conditions, is that deeper layers should consist of fewer latent variables, i.e., 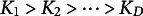 (see Theorems 1, 2, and 3 in the Supplementary Material for detailed identifiability conditions).

In traditional CDMs with a single layer of *K* latent attributes, the 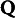 -matrix (Tatsuoka, 1983) is a fundamental component that specifies the relationship between items and the latent attributes. Specifically, 
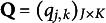
 , where 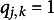 if the item *j* measures the latent attribute *k*, and 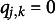 otherwise. Since the edges in a graphical model reflect direct statistical dependencies, 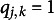 or 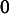 also conveys whether the *k*-th latent node is a parent of the *j*-th observed node. Consequently, the 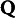 -matrix encodes the bipartite graph structure between the observed and latent layers. Extending this idea to DeepCDMs, with *D* latent layers, requires *D* matrices, denoted as 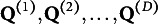 , to capture the dependence relationships between any two adjacent layers. Specifically, 
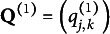
 has size 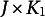 , similar to the single 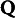 -matrix in traditional CDM, and describes the graph between the observed data layer and the shallowest latent layer. While for 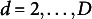 , the matrix 
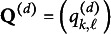
 has size 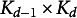 and represents the dependencies between latent variables in the 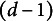 th and *d*th latent layers. The entry 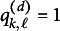 or 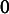 indicates whether the latent variable 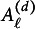 is a parent of 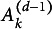 . In this article, we consider the challenging setting of *exploratory* DeepCDMs, where all 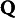 -matrices are unknown and need to be estimated.

Based on the general definition of DAGs in (1) and the DeepCDM setup, the *joint distribution* of all variables, including the latent ones, is given by 
(2)

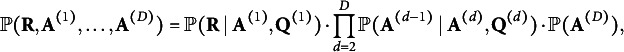



(3)

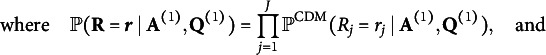



(4)

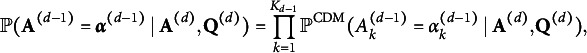

where 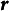 represents an observed response pattern and 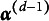 represents a latent pattern for the 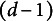 th latent layer. The superscript “CDM” in the conditional distributions of (3) and (4) indicates that the conditional distribution within each layer of the generative process adheres to a CDM. By marginalizing out all latent layers 
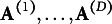
 in (2), we obtain the marginal distribution of the observed response vector 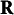 : 
(5)

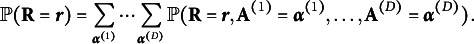



This work focuses on binary observed and latent variables, where 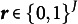 and 
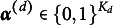
 . Each observed variable reflects whether a response is correct or incorrect, while each latent variable indicates the presence or absence of a specific skill or higher-level attribute. Similar to traditional CDMs, the latent variables 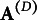 in the deepest layer of a DeepCDM are modeled using a categorical distribution: 
(6)

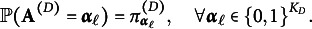

Here, 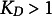 . The proportion parameters 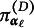 are subject to the constraint 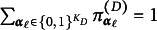 . With this, we complete the specification of a general DeepCDM.

We next discuss the distinction between the proposed DeepCDM and the attribute hierarchy method (AHM; Gierl et al., 2007; Templin & Bradshaw, 2014). Both approaches aim to capture dependencies among latent attributes. However, they differ fundamentally in how such dependencies are represented and inferred. In the AHM framework, hierarchies are defined as deterministic prerequisite relations among attributes within a single latent layer—for instance, mastering attribute *B* is a prerequisite for mastering attribute *A*. These relations constrain admissible attribute profiles to those consistent with the specified hierarchy, embedding learning sequences informed by substantive or curricular theory. In contrast, DeepCDM introduces a probabilistic hierarchy across multiple latent layers. Rather than specifying logical prerequisites within one layer, it models how attributes at one layer are statistically explained by latent traits at a deeper and higher-order layer. Higher-order traits capture shared variance among lower-level attributes, enabling a data-driven hierarchical representation learned directly from data. Overall, these distinctions indicate that AHM and DeepCDM reflect different modeling perspectives and are therefore suited to different types of applications.

### 2.2 Specific examples of DeepCDMs

This section presents concrete examples of DeepCDMs that fall under the general framework outlined in Section 2.1. For notational convenience, we also denote the observed response layer 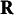 as 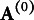 , enabling a unified expression for the layerwise conditional distributions: 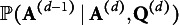 for 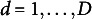 . We then define specific DeepCDM variants according to the diagnostic model adopted for each layerwise conditional.

Example 1 **(Main-effect DeepCDMs).**

We use the term “Main-effect DeepCDMs” to refer broadly to DeepCDMs in which each layerwise conditional distribution follows a main-effect diagnostic model. In this setup, the probability that 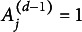 is governed by the main effects of its parent attributes, modeled via a link function 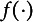 : 
(7)

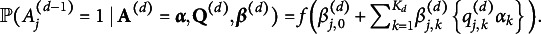

Here, 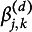 is nonzero only when 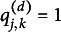 . When *f* is the identity function, Equation (7) reduces to the additive CDM (ACDM; de la Torre, 2011). If *f* is the inverse logit function, Equation (7) gives a logistic linear model (LLM; Maris, 1999).

Example 2 **(All-effect DeepCDMs).**

We refer to DeepCDMs in which the layerwise conditionals follow an all-effect diagnostic model as “All-effect DeepCDMs.” In an all-effect diagnostic model, the probability of 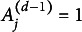 depends on both the main effects and all possible interaction effects of the parent attributes: 
(8)

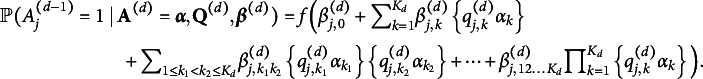

Similar to the main-effect model, not all 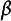 -coefficients above are needed. If 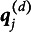 , the *j*-th row of 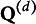 , contains 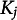 entries of “1,” then 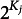 parameters are required in (8). With the identity link function, (8) defines the generalized DINA model (GDINA; de la Torre, 2011), while the inverse logit function yields the log-linear CDM (LCDM; Henson et al., 2009).

Example 3 **(DeepDINA).**

The DINA model can be regarded as a special case of the all-effect CDM, where only the highest-order interaction term among the required attributes is retained, and all lower-order effects are constrained to zero: 
(9)

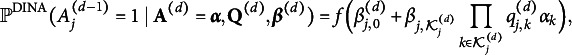

where 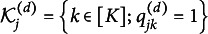 denotes the set of attributes measured by item *j*. The model assumes that students are capable of an item only if they master all required attributes for that item. So, 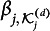 is the only non-zero coefficient for item *j* in layer *d*.

One can also specify a DeepDINO model, a specific type of DeepCDM where the DINO model is used to model each latent layer (Gu, 2024). Due to the duality between DINA and DINO, the identifiability and algorithm applicable to DeepDINA are also applicable to DeepDINO. Therefore, we do not introduce it here and refer readers to Gu (2024) for details.

Example 4 **(Hybrid DeepCDMs).**

A key strength of the DeepCDM framework is its flexibility in allowing different diagnostic models (e.g., DINA, main-effect, and all-effect) to be applied across various layers. This is referred as *Hybrid DeepCDMs*, which strike a balance between model complexity and parsimony, offering flexibility in designing diagnostic models based on specific needs. For instance, in practical scenarios, the most general all-effect diagnostic model may be used at the bottom layer to model how fine-grained attributes affect the observed responses, while simpler models like main-effect or DINA could be applied in deeper layers to enhance interpretability and reduce complexity.

As demonstrated earlier, only particular coefficients, determined by the 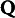 -matrices and the specified measurement models, in the generating DeepCDM should be non-zero. However, since all 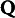 -matrices, 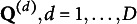 , are unknown, the sparsity pattern of the coefficient vectors is also unknown. Therefore, we assume all coefficients in the model as unknown and estimate them by maximizing a regularized log-likelihood. The 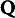 -matrices can then be inferred by identifying the non-zero coefficients in 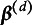 , 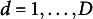 . We defer the details of the mechanism for identifying the entries 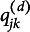 to Section 3.2.

### 2.3 DeepCDMs as deep generative models

As previously mentioned, the DeepCDM framework can be viewed as an adaptation of DGMs for psychometrics and educational measurement, where additional structural constraints are introduced to enable diagnostic feedback. Exploratory DeepCDMs share architectural similarities with several existing DGMs. For example, when the activation function 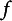 is defined as the inverse logit, DeepCDMs resemble DBNs (Hinton et al., 2006) with binary-valued hidden units. However, a key structural difference lies at the top of the network: DBNs assume an *undirected* graph between the top two layers—forming a restricted Boltzmann machine (RBM)—while DeepCDMs adopt a *fully directed*, top-down architecture across all layers. This design enables DBNs to use a heuristic greedy layer-wise pretraining procedure based on contrastive divergence, a technique specific to training undirected models, such as RBMs (Hinton & Salakhutdinov, 2006; Hinton et al., 2006). Consequently, such training strategies are not directly applicable to DeepCDMs due to its directed nature, which is more interpretable for modeling hierarchical skill generation.

DeepCDMs also share a top-down generative structure with DEFs (Ranganath et al., 2015), an unsupervised framework using exponential family distributions to model each layer’s conditional distribution. DEFs aim to capture compositional semantics through hierarchical latent representations. However, DEFs rely on black-box variational inference with neural network-based posterior approximations, which prevent recovery of interpretable parameters, such as 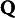 -matrices, and thus cannot provide individualized diagnostic feedback, a central aspect of cognitive diagnosis.

Another related framework is the deep discrete encoders (DDEs; Lee & Gu, 2025), a DGM designed for rich data types with discrete latent layers. While DDEs and DeepCDMs share architectural and identifiability properties, their goals differ. DDEs aim to address machine learning concerns like overparameterization and lack of interpretability, constructing general-purpose identifiable DGMs. In contrast, DeepCDMs are specifically designed for psychometrics, with each adjacent pair of latent layers constituting a CDM. This structure allows for diagnostic-specific measurement assumptions, addressing varied diagnostic goals and enhancing usability in real-world assessment settings.

Taken together, these distinctions highlight the unique features of exploratory DeepCDMs over existing DGMs. While most DGMs prioritize data generation or predictive performance and typically lack identifiability and sparsity constraints, DeepCDMs refocus the modeling effort on offering reliable individualized diagnostic feedback and discovering hierarchical latent skill structures. Furthermore, by enforcing sparsity in the coefficient matrices to reflect item–attribute relationships, DeepCDMs provide valuable insights into test design—an essential feature for practical use in educational and psychological assessment, often overlooked in classical DGM frameworks.

### 2.4 Identifiability

As noted earlier, a key strength of DeepCDMs lies in their formal identifiability guarantees, which apply to both confirmatory and exploratory settings (Gu, 2024). These results are detailed in the Supplementary Material. In brief, the identification conditions impose explicit structural constraints on the between-layer 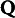 -matrices, offering practical guidance for model design and implementation. Although the specific conditions vary across diagnostic models, they consistently require an increasingly *shrinking latent structure* for deeper layers. That is, the number of latent variables typically decreases with depth, often subject to constraints, such as 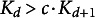 for some constant 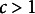 depending on the model. This hierarchy reflects the principle of *statistical parsimony* in DeepCDMs. For instance, in a two-layer DeepDINA model with 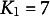 and 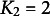 , the number of nonzero parameters is 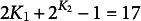 , compared to 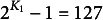 in a saturated attribute model without higher-order latent structure. Such substantial reductions in complexity make DeepCDMs especially attractive for applications with fine-grained latent attributes and limited sample sizes. In exploratory settings, while all parameters must be estimated, the identifiability conditions naturally promote sparsity in the true generating model, facilitating parameter recovery and interpretation of the latent attributes.

A central insight underlying these proofs is that the identifiability of DeepCDMs can be established in a layer-by-layer fashion, proceeding from the bottom (shallow) layer to the top (deepest) layer. This approach is justified by the directed nature of the graphical model and the discreteness of latent variables. Two core ideas facilitate this stepwise identifiability. First, in a multi-layer directed graphical model with only top-down connections between adjacent layers, marginalizing out deeper latent layers yields a restricted latent class model (RLCM) (Gu & Xu, 2020; Xu, 2017). Once the distribution of the shallowest latent layer is identified through this RLCM, it can be treated *theoretically as if observed* for identifying the next deeper layer. Second, identifiability in RLCMs holds for any marginal distribution of latent attributes, provided the 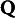 -matrix meets specific conditions. This property allows identifiability to propagate upward through layers, even when deeper latent variables introduce complex dependencies.

## 3 Proposed estimation algorithms

In this section, we propose a novel layer-wise EM algorithm for estimating exploratory DeepCDMs. We begin by introducing some notation. Let 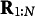 denote the response data matrix of size 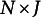 , representing the observed responses of *N* students to *J* items. Let 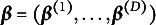 and 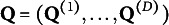 denote the sets of continuous parameters and 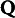 -matrices across all layers, respectively. Let 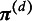 denote a vector composed of the probability mass function 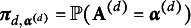 for all 
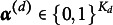
 , 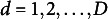 . The parameters to be estimated include all continuous parameters in 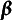 , all 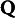 -matrices in 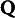 , and the proportion parameter 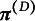 for the deepest latent layer. Directly maximizing the marginal log-likelihood to estimate the 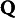 -matrices is computationally prohibitive, even when the number of layers *D* and the dimensionalities 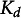 ( 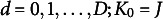 ) are of moderate size. This challenge arises from the need to search over an enormous space of possible 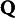 -matrix configurations—specifically, 
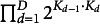
 combinations—each requiring evaluation of a profile likelihood. To avoid this combinatorial burden, we instead frame 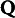 -matrix estimation as a structured model selection task, addressed via a regularized likelihood approach that encourages sparsity in the parameter space (Chen et al., 2015).

The regularized marginal log-likelihood is given by 
(10)

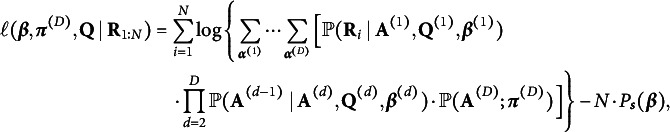

where the 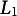 penalty function 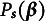 enforces sparsity across layers and is defined as 
(11)

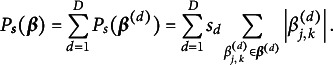

Here, 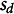 denotes the regularization parameter for layer 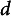 , and each 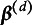 represents the set of model-specific coefficient parameters at that layer, with its structure determined by the chosen measurement model (e.g., main-effect, all-effect, or DINA), as detailed in Section 2.2.

The nested summation over multiple layers of latent attributes in (10) renders direct optimization infeasible. While the classical EM algorithm offers a principled framework for estimating exploratory DeepCDMs, it is not without limitations. In practice, its effectiveness can be hindered by the model’s structural complexity and the high dimensionality of the parameter space. These challenges, stemming from the layered latent architecture and the combinatorial nature of 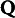 -matrix estimation, can increase sensitivity to initialization and compromise scalability and stability. A detailed discussion of these shortcomings is provided in Section 3.4. These considerations motivate our development of the layer-wise EM algorithm, introduced in the following section.

In the remainder of this section, we first introduce the classical EM algorithm and briefly discuss its limitations in Section 3.1. The proposed layer-wise EM algorithm is presented in Section 3.2, followed by the initialization strategy in Section 3.3. Section 3.4 highlights the advantages of the layer-wise EM over the classical EM algorithm. Section 3.5 discusses how the layer-wise concept connects to broader principles and algorithms, including identifiability derivation and related methods for DGMs. Finally, Section 3.6 discusses the extension of the layer-wise EM to the confirmatory DeepCDM setting.

### 3.1 The EM algorithm

Let 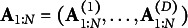 denote the set of latent variables, i.e., the attribute profiles of the *N* students across *D* latent layers. The complete data log-likelihood is: 
(12)

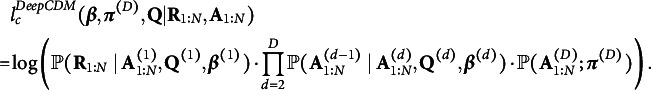



Let 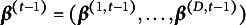 , 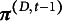 , and 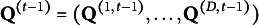 denote the estimates of 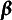 , 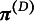 , and 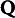 obtained at iteration 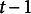 . In each iteration *t* of the EM algorithm, the following two steps are performed:

*E-Step*: Compute 
(13)

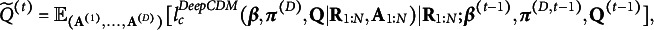

where the conditional expectation is with respect to 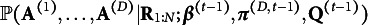 .

*M-Step*: Update 
(14)

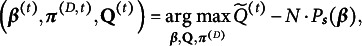

where 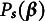 is defined in Equation (11).

For each 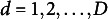 , define 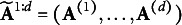 , which denote the latent variables shallower than the 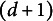 th latent layer. Define 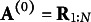 and 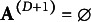 . According to the conditional independence, the expectation computation in the E-step can be re-expressed as 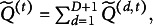 with 
(15)

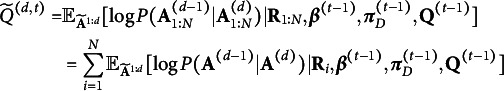

That is, 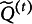 is decomposed as a summation over layers *d* and individuals *i*, where each term is the conditional expectation of 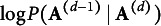 with respect to the partial posterior distribution 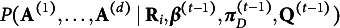 , which we denote by 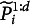 for brevity.

Accordingly, the optimization in the M-step can be broken into the following parts: 
(16)

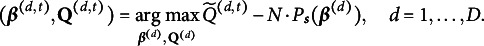



(17)

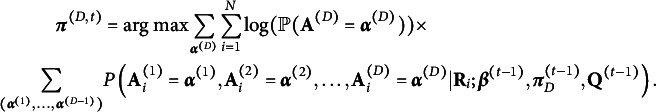

This decomposition enables the parameters at each layer to be updated via their corresponding optimization problems in (16) and (17), thereby improving the tractability of the M-step. Next, we further look into the 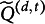 functions. Recall that 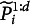 is defined as 
(18)

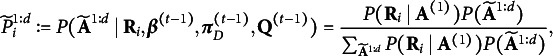

with 
(19)

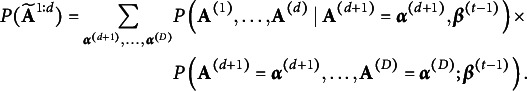



As shown, given the parameters from the previous iteration 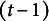 , the distribution 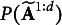 is computed by marginalizing out the deeper latent variables 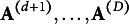 from the joint distribution over all latent variables. This marginal, together with the observed response 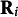 , allows us to evaluate the partial posterior distribution 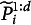 via Equation (18). With 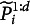 providing the weights for each possible configuration of 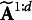 , the conditional expectation in 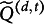 can be computed as a weighted sum over 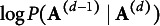 . Specifically, Equation (15) can be written out as 
(20)

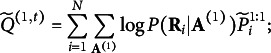

and 
(21)

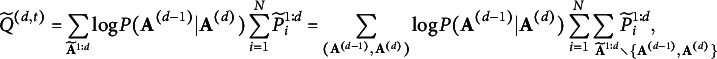

for 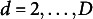 . It turns out that, for each *d*, Equation (16) defines a regularized optimization problem whose objective includes a weighted log-likelihood component over 
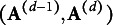
 pairs. In this formulation, 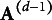 serves as the outcome, 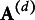 as the predictor, and the data point weights are given by 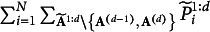 .

We focus on the case where the link function 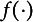 is the inverse logit, as it is the most commonly used choice for CDMs with binary responses. In this setting, the estimation problem corresponds to a generalized linear optimization problem with a logit link. For other choices of 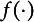 , the problem may fall into the broader categories of linear or generalized linear optimization, depending on the specific functional form. The solution of Equation (17) is that for 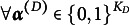 : 
(22)

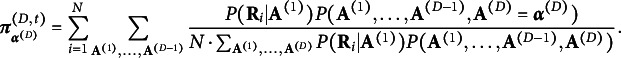

These derivations demonstrate that, due to conditional independence, the EM algorithm for exploratory DeepCDMs is both succinct and transparent. However, its practical feasibility is challenged by several issues—particularly sensitivity to initialization and the accumulation of estimation bias across layers and iterations. To address these challenges, we next propose a layer-wise EM algorithm below.

### 3.2 A novel layer-wise EM algorithm

To elucidate the underlying rationale of the proposed algorithm, we first provide a detailed mathematical derivation of the layer-wise EM procedure. This step-by-step formulation highlights how the algorithm naturally arises from the hierarchical structure of DeepCDMs.

Suppose we have a set of parameters 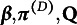 that maximize the regularized marginal log-likelihood in Equation (10). Based on the generative formulation of DeepCDMs, we can marginalize out the deeper latent variables 
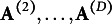
 to derive the implied distribution of the bottom-layer attributes: 

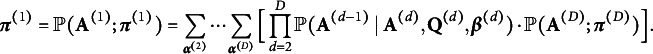

This recursive marginalization induces a set of shallow-layer parameters 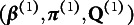 , which, when substituted into the original model, must also maximize a re-expressed form of the target function: 

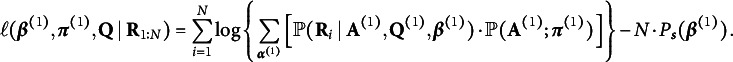

This observation forms the conceptual basis of our proposed layer-wise EM algorithm. Rather than estimating all parameters jointly over the entire deep latent architecture, we decompose the problem into a sequence of simpler subproblems, each involving a one-layer structure, and solve them in a bottom-up manner using EM. Although the resulting algorithm appears intuitive, it is grounded in a rigorous use of the model’s generative structure. Specifically, each layer-wise step leverages the most reliable information from its immediate lower layer—either in the form of estimated distributions or pseudo-observations—making the estimation process both computationally efficient and statistically reliable.

Focusing on the first layer ( 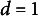 ), this decomposition implies that the estimates 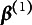 and 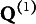 obtained by maximizing Equation (10) are identical to those obtained under a standard one-layer CDM. Once these first-layer parameters are estimated, the task reduces to estimating the parameters for the remaining 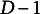 latent layers. Unlike the first layer, however, there are no observed realizations of the latent variable 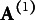 . Let 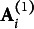 denote the 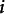 -th row of 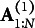 . A straightforward way to impute 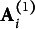 is via the maximum a posteriori (MAP) estimate: 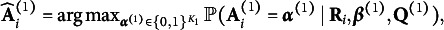 which depends on both the likelihood 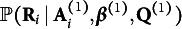 and the prior 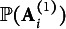 . Alternatively, one can sample from the estimated marginal distribution 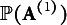 to generate pseudo-observations for the next layer. Compared to MAP, this sampling-based approach introduces less bias and leads to more reliable parameter estimation in deeper layers, particularly during initialization. With these pseudo-observations in hand, the remaining 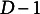 layers form a structurally similar DeepCDM, and the same one-layer EM algorithm can be recursively applied to estimate 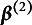 and 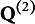 , and so on, until the top layer is reached.

The above derivation implies that the estimator obtained from the layer-wise procedure is statistically equivalent to the estimator obtained by jointly maximizing the full DeepCDM likelihood, and thus achieves the same asymptotic efficiency. This procedure offers practical computational advantages, as discussed in Section 3.4. Although the estimation at layer *d* uses only the latent variables from the immediately deeper layer 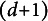 , this does not discard the dependencies encoded in all deeper layers. Indeed, that information is summarized in the aggregated prior 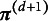 , which captures the deeper dependency structure and allows the procedure at layer *d* to focus on recovering the relationship between 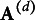 and 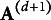 . By iterating this process upward through the hierarchy, the estimated parameters 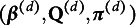 across all layers collectively recover the full cross-layer dependence structure.

Algorithm 1 summarizes the proposed layer-wise EM algorithm. Proceeding from the bottom up, the algorithm estimates model parameters layer by layer. For each layer 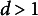 , Step 1 imputes the missing data 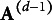 by drawing samples from the estimated marginal distribution 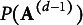 , which is computed in Step 3 of the previous layer using Equation (23). In contrast, for the first latent layer ( 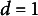 ), Step 1 is not required because the observed response data 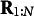 (i.e., 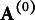 ) serve directly as the input. Using the initial values obtained in Step 2, Step 3 then applies a one-layer EM algorithm with 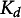 attributes to estimate the parameters 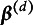 , 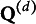 , and 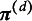 . The initialization procedure is introduced separately in Section 3.3. In Step 3, each M1-step is solved using the coordinate descent algorithm of Friedman et al. (2010), known for its flexibility and effectiveness in handling regularized optimization problems. The algorithm continues this layer-wise procedure until reaching the deepest layer 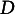 .

**Figure:**
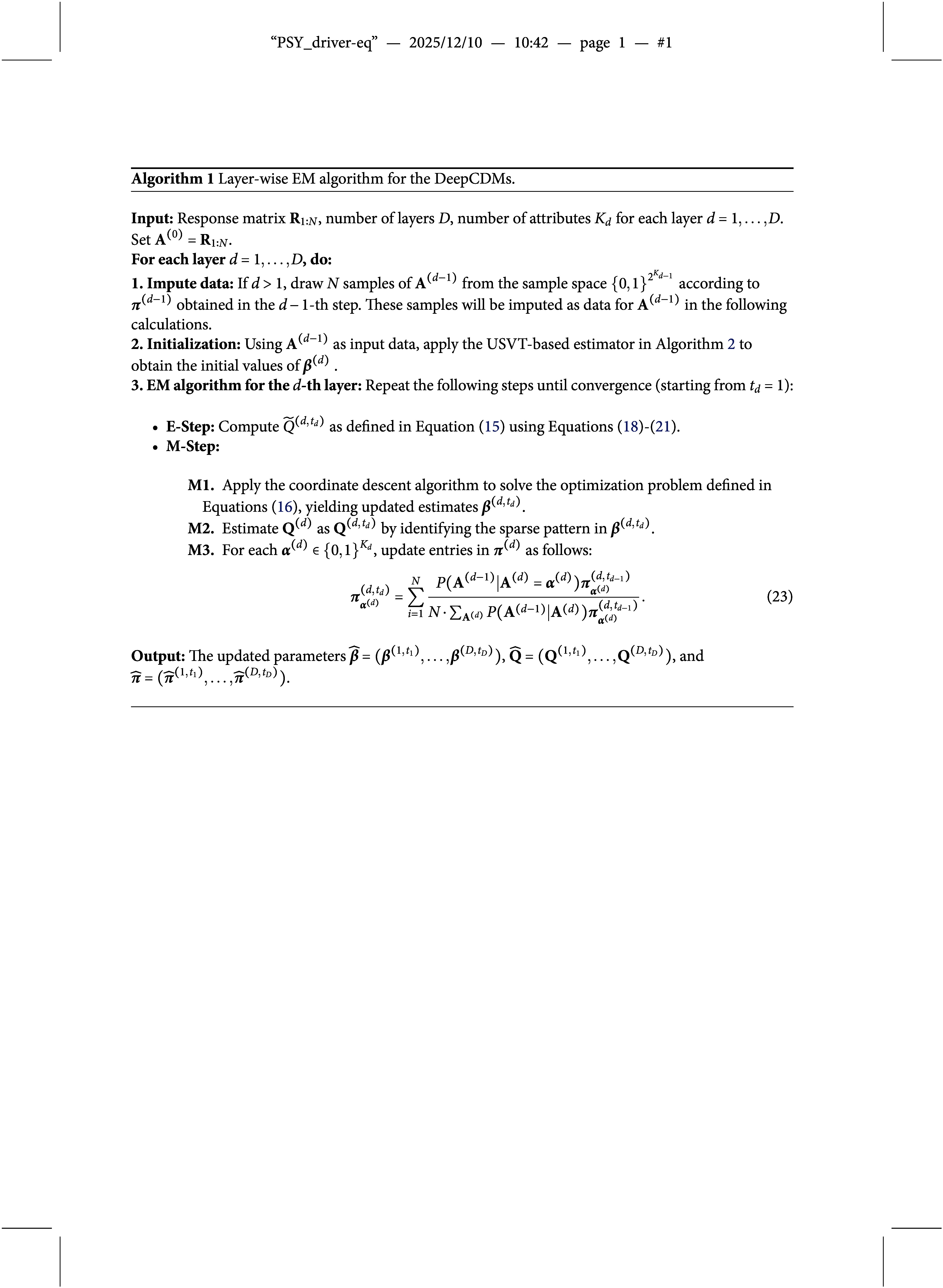

In M2-step, the mechanism for identifying the entries 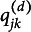 in 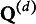 varies across different measurement models, according to the measurement model utilized in layer *d*. For the *main-effect*-model, 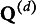 can be recovered using the rule 
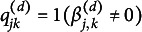
 , where 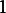 is the indicator function. For the *all-effect* model, theoretically, each row of 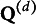 should be identified by the highest-order non-zero coefficient. Specifically, if 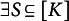 such that 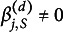 and 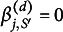 for all 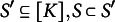 , then 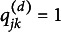 for 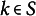 ; otherwise, 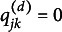 . However, this strict rule may not always be applicable because some estimated 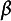 -coefficients may be close to zero but not exactly zero due to residual regularization noise. In practice, a more effective approach is either to choose the largest non-zero interaction coefficient or to truncate the coefficients before identifying 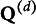 . For the latter approach, we recommend practitioners set the truncation thresholds based on the general magnitude of their estimated coefficients. For the *DINA model*, since there should be only one non-zero coefficient for each item 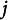 , the largest non-zero interaction coefficient can be selected, and the corresponding 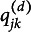 can be identified as equal to one. We note that seemingly poor 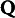 -matrix recovery may appear if such residual regularization noise is not properly addressed, as it can obscure the algorithm’s underlying recovery performance.

### 3.3 Initialization algorithm

As shown in Algorithm 1, the initialization of our DeepCDM method is performed sequentially in *D* steps, where parameters from the *d*-th latent layer are initialized after estimating the 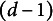 -th layer. This approach leverages the information from the estimated distribution 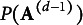 , obtained during the fitting of the 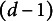 -th layer, to provide better initial values for the *d*-th layer. Furthermore, by imputing realizations of 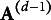 sampled from the estimated 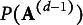 , the initializations of all layers reduce to the problem of initializing a one-layer CDM. Specifically, the response data 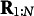 are used for the first layer ( 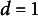 ), while realizations of 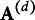 (i.e., the generated pseudo-samples) are used for subsequent layers ( 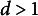 ). For exploratory DeepCDMs, good initial values should not only be close to the true values but also exhibit a sparse structure similar to the true ones. To achieve this, we apply a USVT-based method (Chatterjee, 2015; Zhang et al., 2020) to estimate the design matrix, followed by a Varimax rotation to promote sparsity. USVT captures the dominant low-rank structure, while Varimax produces a sparse loading pattern that informs the initial 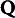 -matrix. This combination provides informative starting values tailored to the exploratory framework of DeepCDMs. The initialization procedure for each layer *d* is detailed in Algorithm 2.

**Figure:**
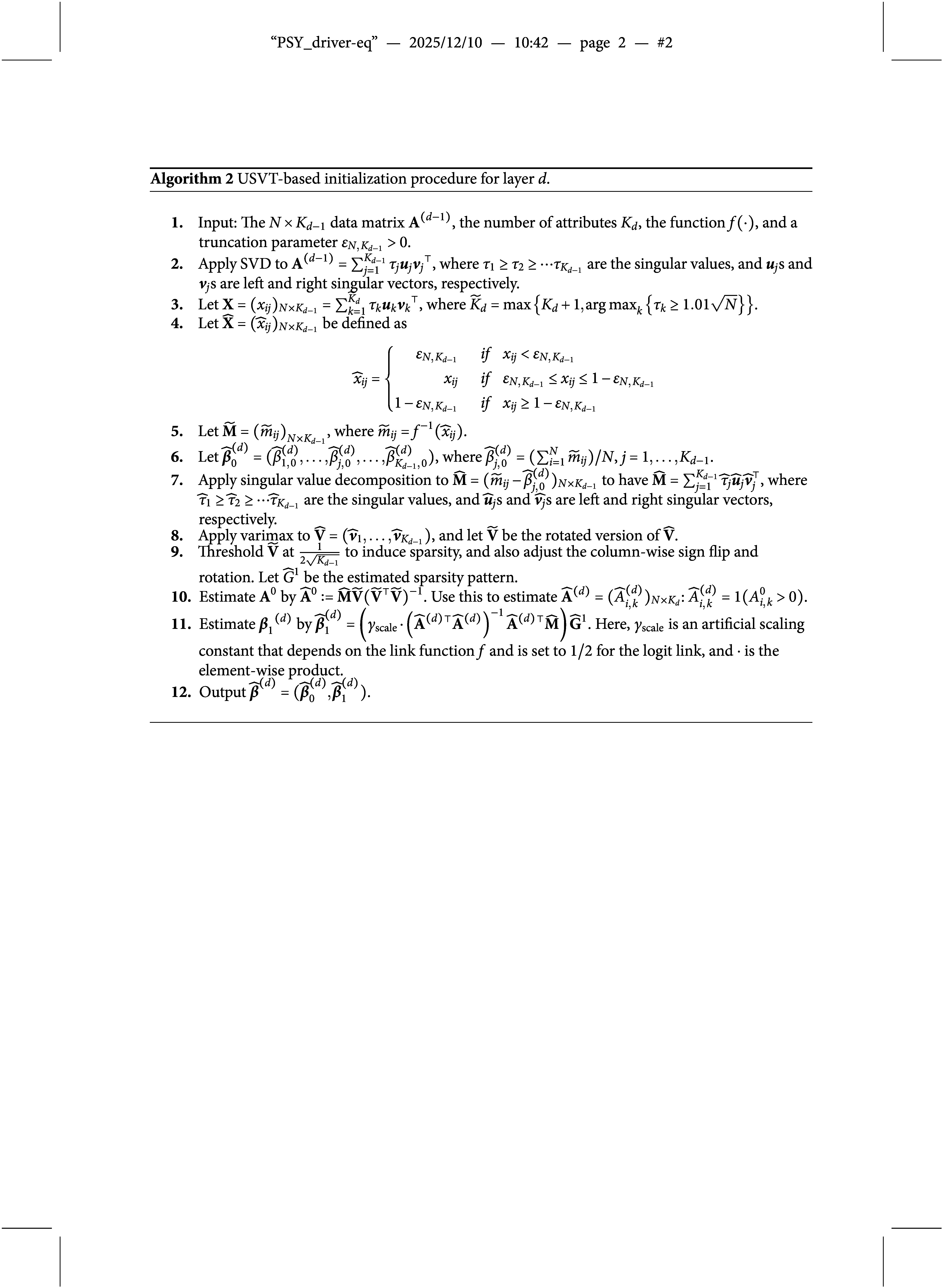

Algorithm 2 applies SVD twice. The first application, combined with the inverse transformation (Steps 2–5), is used to denoise and linearize the data. The second application of SVD (Steps 6 and 7) is performed on the linearized data. Since SVD solutions are rotation-invariant while the true coefficient matrix 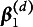 is sparse, we address this invariance by seeking a sparse representation of the denoised data that is consistent with the model’s identifiability conditions and expect it to share the same sparsity pattern as 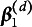 . We apply the Varimax rotation for this purpose in Step 8, as it induces sparse loadings and possesses well-established statistical inference properties (Rohe & Zeng, 2023). To further refine sparsity, a thresholding step is applied to eliminate near-zero loadings resulting from sampling variability (Step 9). Rather than using the conventional threshold 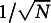 , we adopt a more relaxed threshold 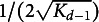 , which is more adaptive to the multilayer structure of DeepCDMs—since pseudo-data in deeper layers tend to be noisier, a more relaxed threshold helps mitigate such effects. If the rotated Varimax loadings were used directly to estimate 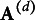 and 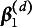 , the estimates could differ from the true ones in scale, as Varimax does not constrain the scaling of rows or columns. For 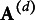 , although this scaling issue remains, the binary structure can still be identified by noting that 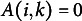 if and only if 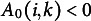 in Step 10. We then exploit the discreteness of the latent variables in 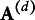 to rescale 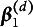 in Step 11, yielding correctly scaled estimates. This initialization procedure is non-iterative, making it computationally efficient. Moreover, it avoids convergence issues and possesses favorable statistical consistency properties (Zhang et al., 2020).

Additionally, as pointed out by one reviewer, one can also directly apply the Varimax rotation to the matrix 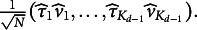 A simulation study comparing the performance of these two approaches is reported in the Supplementary Material.

### 3.4 Advantages of layer-wise EM compared to EM

As discussed in Hinton et al. (2006), an effective way to learn complex models is by sequentially combining simpler models. The layer-wise EM algorithm applies this principle by decomposing the optimization into manageable subproblems, each targeting one layer at a time. This strategy helps overcome key challenges faced by the classical EM algorithm when applied to DeepCDMs.

One major challenge in applying the classical EM algorithm to DeepCDMs is the difficulty of initialization. The presence of multiple nonlinear latent layers creates a highly nonconvex optimization landscape, often with an exponential number of local optima. As a result, EM is sensitive to starting values—poor initialization can easily lead to convergence at suboptimal local maxima of the penalized log-likelihood function. In the classical EM algorithm, initial values for all parameters across all layers must be specified simultaneously. As the model depth increases, this becomes increasingly challenging, even for moderately deep architectures, due to compounded uncertainty and parameter interactions across layers. In contrast, our layer-wise EM algorithm addresses initialization sequentially by solving one-layer CDMs one at a time. At each stage, it initializes the parameters of the current layer using either observed responses or sampled latent attributes from the estimated marginal distribution informed by the shallower layers. Since these samples are based on estimated proportion parameters which are proved to be identifiable, the initialization for deeper layers is more stable and reliable.

Another major issue of the classical EM algorithm is the accumulation of estimation bias as the algorithm progresses through multiple layers. As the number of latent layers 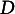 increases, the computation of quantities like 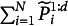 for 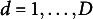 becomes more error-prone. These quantities play a crucial role in the M-step, and inaccuracies in their estimation directly affect parameter updates. Furthermore, the iterative nature of the classical EM algorithm introduces a cyclic dependency across all layers, where errors at one layer can propagate forward and backward, reinforcing one another over iterations. This compounding effect often leads the algorithm to converge to suboptimal local maxima, even when reasonably good initial values are provided. In contrast, our proposed layer-wise EM algorithm mitigates this issue by breaking the dependency cycle. Parameters are estimated sequentially from the bottom layer up, so each layer 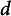 is only influenced by the estimates from shallower layers 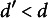 . Although some bias may still accumulate, it originates solely from estimation errors in previous layers—not from compounded initialization errors across all layers. This directional, non-cyclic structure significantly reduces the accumulation of error and enhances the robustness of the estimation process.

### 3.5 Connections to broader principles and algorithms

#### 3.5.1 Connections between the layer-wise EM and identifiability

Interestingly, the derivation of the layer-wise EM algorithm aligns with and supports the technical insights in the identifiability proofs of DeepCDMs. The identifiability results in the Supplementary Material and in Gu (2024) demonstrate that the identifiability of a DeepCDM can be examined and established in a layer-by-layer manner, proceeding from the bottom up. This follows from the probabilistic formulation of the directed graphical model and the discrete nature of the latent layers. For each layer 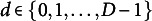 , as long as 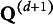 satisfies the corresponding identifiability conditions for the one-layer CDM implied by 
(24)

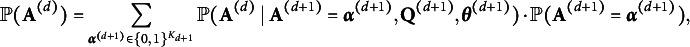

the parameter set 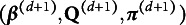 is identifiable. The identifiability of the marginal distribution of 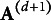 (i.e., 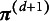 ) allows it to be treated theoretically as if it were observed when examining the identifiability for 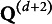 , 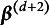 , and the marginal distribution of 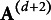 . Starting from the observed data layer ( 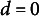 ) and proceeding one layer at a time, the identifiability of DeepCDMs can thus be inductively established.

This layer-wise identifiability rationale supports the procedure of generating samples of 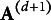 when estimating parameters of the 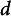 -th layer, for each *d*. Specifically, the identifiability of the model at layer 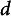 , as shown in Equation (24), implies that the distribution 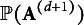 , from which samples of 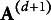 are drawn, can be uniquely identified. This theoretical guarantee justifies treating the sampled 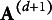 as observed data in the EM algorithm. The same procedure is applied recursively to the remaining 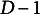 layers.

#### 3.5.2 Connections to related algorithms for DGMs

The idea of training deep models in a layer-wise fashion has been widely explored across the deep generative modeling literature, and our layer-wise EM algorithm draws on this foundational principle. For instance, in DBNs, greedy layer-wise pretraining trains each layer locally as an RBM using contrastive divergence (Hinton et al., 2006). This approach improves optimization stability and scalability, particularly in deep models where global joint training is challenging. While DeepCDMs differ from DBNs in having a fully directed architecture and an emphasis on interpretability and identifiability, our method similarly decomposes model training into a sequence of tractable subproblems. DEFs (Ranganath et al., 2015) also adopt a hierarchical top-down structure and rely on recursive variational inference across layers. Although our inference uses EM rather than variational methods, both approaches share the advantage of progressing layer by layer, with each layer conditioned on information derived from the previous one. Our idea also resonates with the framework of modular Bayesian learning (Joshi et al., 2009; Segal et al., 2005), in which large models are decomposed into smaller, interpretable modules that can be estimated sequentially. By aligning with these broader principles, the proposed layer-wise EM adapts a widely used idea—local, modular, progressive learning—for the structured and interpretable setting of cognitive diagnosis, where both identifiability and individualized feedback are essential.

### 3.6 Extending to confirmatory DeepCDMs

The layer-wise EM algorithm can be easily modified to fit confirmatory DeepCDMs, which assume all 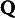 -matrices are known. In this case, each M-step solves the following optimization: 
(25)

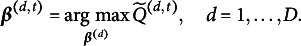

Compared to Equation (16), the terms 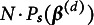 are dropped, as all the 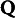 -matrices are known and do not need to be estimated. This makes the confirmatory case simpler than the exploratory case we have focused on. The layer-wise algorithm in Algorithm 1 can be readily used for parameter estimation, incorporating the known 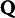 -matrices during the implementation of coordinate descent in the M-step.

The adaptation of the layer-wise EM algorithm for the confirmatory case is also a novel contribution, representing the first EM-type algorithm for confirmatory DeepCDMs. Unlike the Bayesian approach in Gu (2024), which fits models using an MCMC algorithm that can involve slower convergence and the need for prior specification, the layer-wise EM algorithm offers a computationally more efficient alternative by directly seeking the maximum likelihood estimator. We note that the layer-wise EM method could also be extended to incorporate prior distributions for computing maximum posterior estimates, should rich prior information be available, following the approach outlined in Gu (2024).

## 4 Simulation studies

In this section, we conduct simulation studies to evaluate the performance of the proposed layer-wise EM algorithm for exploratory DeepCDMs. Specifically, we consider a three-layer DeepCDM ( 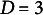 ) with the configuration 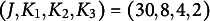 , which represents a challenging scenario due to the model depth and the need to estimate all three unknown 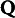 -matrices across different layers. Three different measurement models are considered for the shallowest layer ( 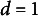 ): the main-effect model, the all-effect model, and the DINA model. The more parsimonious main-effect model is used to model the two deeper layers ( 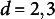 ). We denote these three simulation cases as the Main-effect case, All-effect case, and DINA case, respectively. Under each case, three sample sizes— 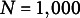 , 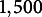 , and 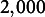 —are considered. The true 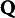 -matrices are specified in Equation (26) and satisfy the minimal identifiability conditions required for DeepCDMs: 

(26)

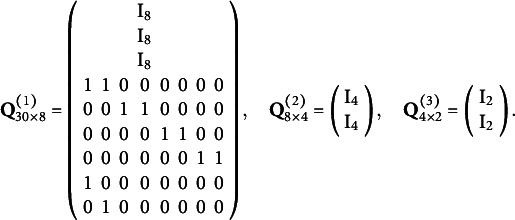



The USVT-based estimator described in Algorithm 2 is used for initialization. The coordinate descent algorithm is implemented using the R package *glmnet* (Hastie et al., 2021) for optimization. The true coefficient values of 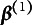 , 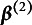 , and 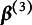 are presented in the following sections for each simulation case, where 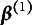 is set to be larger than those of the two deeper layers, 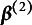 and 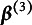 . In CDM, larger coefficient values indicate a stronger relationship between latent attributes and responses. Therefore, by specifying smaller coefficients for the deeper layers, we introduce more randomness into this relationship. This design reflects the increased uncertainty and complexity involved in mastering higher-level cognitive attributes in psychological or educational assessments. In this simulation, the convergence thresholds are set as 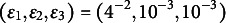 . A more relaxed threshold is adopted for the first layer to accommodate its relatively larger number of parameters, as this layer involves both a larger 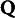 -matrix ( 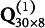 ) and larger coefficient magnitudes in 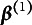 compared to the deeper layers. The specification of 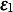 follows the same order of magnitude as the threshold of 0.06 used in sparse exploratory MIRT models in Li et al. (2025). The specification of these thresholds is guided by the model scale and the numerical characteristics observed at each layer during estimation, with the aim of balancing estimation precision and computational efficiency. In practice, practitioners may adjust these thresholds according to the scale of 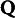 and the magnitude of estimated parameters, or employ smaller values for stricter convergence when computational resources permit. For each simulation case, 100 independent replications are conducted. In each replication, the layer-wise EM algorithm is applied to fit the model across a range of regularization parameters, and the one that yields the smallest BIC value is selected for final model fitting. The specific regularization sequences are provided in the Supplementary Material. Root mean-squared errors (RMSE) and absolute biases (aBias) are computed to assess estimation accuracy.

Note that the true parameter values differ in magnitude across the three layers, making the RMSE and aBias values not directly comparable. To address this, we report RMSE and aBias for two additional metrics that evaluate the performance of the layer-wise EM algorithm from different perspectives and provide indices that are comparable across layers. The first index is the latent class proportion distribution 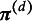 , whose parameter space is defined as 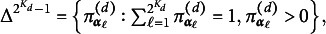 where 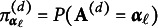 and 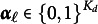 for each layer 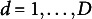 . This metric assesses how accurately the model recovers the true distribution of latent attributes at each layer. The second index is the correct response probability for each layer *d*, denoted as 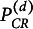 , and defined by Equations (7)–(9) according to the measurement model employed. This metric evaluates how well the model predicts correct responses at the population level for each layer. In the case of a single latent layer, this probability is commonly represented in the literature as 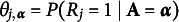 .

### 4.1 Simulation studies for main-effect DeepCDMs

Throughout this section, let 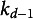 and 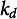 be the indices for the 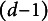 -th and *d*-th layers, respectively. For the main-effect models, the coefficients 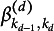 are specified as 
(27)

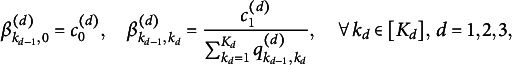

where 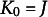 , and the constants 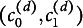 are set to 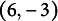 , 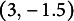 , and 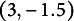 for 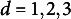 , respectively. The RMSE and aBias are reported in Table 1. All indices decrease as the sample size increases, indicating improved estimation accuracy. For each sample size, the estimation accuracy is lower in the deeper layers than in the shallower layers, as reflected by the values of 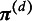 and 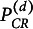 . This result is intuitively reasonable, as estimating deeper layers is fundamentally more challenging due to stochastic latent layers. In addition, all RMSE and aBias values remain reasonably small, providing empirical support for the identifiability of the model.

**Table 1:** RMSE and aBias for the main-effect DeepCDM

			RMSE			aBias		
Measurement model	*N*	Layer (*d*)	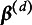	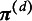	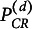	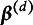	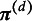	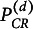
		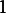	0.408	0.0190	0.022	0.355	0.0015	0.015
	1,000	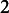	0.189	0.0220	0.052	0.127	0.0170	0.034
		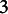	0.459	0.0690	0.075	0.319	0.0540	0.056
			Computation time (min): 4.13			Iterations: 78.8		
		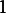	0.338	0.0016	0.021	0.334	0.0013	0.015
	1,500	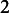	0.186	0.0205	0.047	0.121	0.0150	0.034
		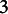	0.416	0.0510	0.074	0.306	0.0385	0.052
			Computation time (min): 4.48			Iterations: 75.86		
		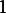	0.302	0.0014	0.015	0.297	0.0011	0.013
Main-effect	2,000	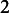	0.179	0.0170	0.044	0.113	0.0138	0.030
		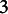	0.372	0.0490	0.055	0.243	0.0385	0.042
			Computation time (min): 13.67			Iterations: 71.81		

To examine the recovery of the 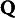 -matrices, we report the proportion of correctly estimated rows and entries in each 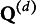 for 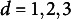 , as shown in Table 2. Note that these indices are not directly comparable across layers at each sample size, as the proportions depend on both the size of the 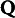 -matrix and the difficulty of parameter estimation. Due to the shrinkage ladder structure, which is supported by the identifiability conditions, the shallower layers contain larger 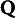 -matrices, making their structures more challenging to recover. However, these layers also benefit from more informative signals, as they are closer to the observed data layer. In contrast, the deeper layers involve smaller 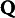 -matrices that are structurally easier to recover, but they suffer from less informative signals due to the greater amount of uncertainty introduced at deeper levels. Despite these differences, when comparing 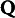 -matrix recovery across sample sizes, it is evident that for each layer *d*, the estimation accuracy of 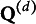 improves as the sample size increases.

**Table 2:** Proportion of correctly recovered rows ( 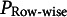 ) and entries ( 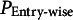 ) for the main-effect DeepCDM

*N*	Proportion	Layer 1	Layer 2	Layer 3
1,000	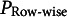	0.490	0.697	0.829
	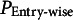	0.932	0.956	0.901
1,500	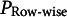	0.548	0.704	0.820
	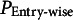	0.945	0.957	0.931
2,000	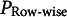	0.582	0.783	0.845
	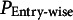	0.947	0.968	0.935

### 4.2 Simulation studies for all-effect DeepCDMs

Denote the true coefficients as 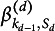 , 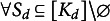 and 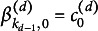 . In the all-effect DeepCDMs case, denote 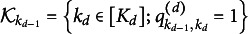 , and 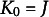 , and the first layer ( 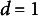 ) is modeled using the all-effect model with the parameters given below: 

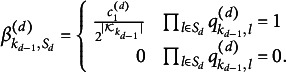

The two deeper layers ( 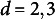 ) are then modeled by the main-effect model, with parameters specified according to Equation (27). The constants 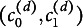 are specified as 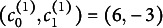 , 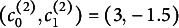 , and 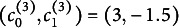 . The 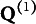 -matrix is recovered by identifying, for each item, the highest-order nonzero interaction coefficient.

The RMSE and aBias values are summarized in Table 3. As expected, all indices decrease with increasing sample sizes, indicating improved estimation accuracy. RMSE and aBias values remain reasonably small across all layers, providing empirical support for the identifiability of the model. To evaluate the recovery of the 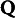 -matrices, we report the proportions of correctly estimated rows and entries in each 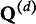 for 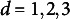 , as shown in Table 4. As in previous cases, these proportions are not directly comparable across layers, as they are influenced by differences in 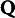 -matrix size and estimation difficulty. Nevertheless, for each layer *d*, the estimation accuracy of 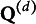 improves as the sample size increases.

**Table 3:** RMSE and aBias for the all-effect DeepCDM

			RMSE			aBias		
Measurement model	*N*	Layer (*d*)	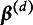	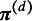	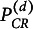	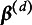	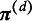	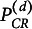
		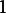	0.486	0.0018	0.025	0.432	0.0015	0.020
	1,000	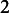	0.197	0.0250	0.047	0.132	0.0198	0.037
		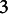	0.660	0.0800	0.095	0.427	0.0600	0.070
			Computation time (min): 32.00			Iterations: 75.78		
		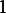	0.428	0.0015	0.021	0.386	0.0012	0.016
	1,500	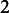	0.191	0.0216	0.044	0.128	0.0175	0.034
		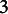	0.451	0.0700	0.073	0.326	0.0520	0.058
			Computation time (min): 25.74			Iterations: 80.15		
		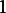	0.389	0.0013	0.017	0.353	0.0010	0.014
All-effect	2,000	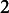	0.144	0.0160	0.033	0.104	0.0130	0.026
		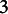	0.300	0.0440	0.051	0.205	0.0350	0.039
			Computation time (min): 63.37			Iterations: 68.76

**Table 4:** Proportion of correctly recovered rows ( 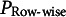 ) and entries ( 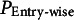 ) for the all-effect DeepCDM

*N*	Proportion	Layer 1	Layer 2	Layer 3
1,000	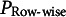	0.808	0.662	0.745
	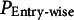	0.986	0.942	0.867
1,500	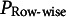	0.852	0.697	0.750
	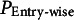	0.990	0.947	0.888
2,000	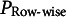	0.878	0.757	0.885
	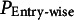	0.993	0.969	0.954

### 4.3 Simulation studies for DINA-effect DeepCDMs

Denote the true coefficients as 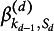 for all 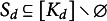 , with 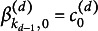 . In DINA DeepCDMs, the first layer ( 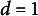 ) is modeled using the DINA formulation where 
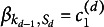
 if 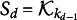 , and zero otherwise, with 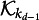 defined in Section 4.2. The DINA model can be viewed as a special case of the all-effect model, in which non-zero coefficients are assigned only to the interaction of all attributes required by the 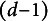 -th unit in each *d*-th layer model. This formulation allows the same estimation framework used for the all-effect model to be applied to the DINA model. The two deeper layers ( 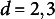 ) are modeled using the main-effect specification, with parameters defined as in Equation (27). The constants 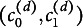 are specified as 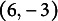 , 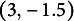 , and 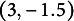 for 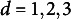 , respectively.

The RMSE and aBias results are reported in Table 5. Again, these values exhibit a clear decreasing trend with increasing sample size, indicating improved estimation accuracy. To assess the recovery of the 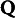 -matrices, Table 6 presents the proportions of correctly estimated rows and entries for each 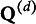 , 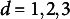 . Overall, for all layers, the estimation accuracy of 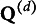 improves as the sample size increases.

**Table 5:** RMSE and aBias for the DINA DeepCDM

			RMSE			aBias		
Measurement model	*N*	Layer (*d*)	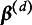	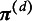	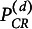	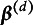	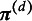	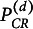
		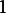	0.436	0.0018	0.022	0.412	0.0015	0.017
	1,000	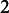	0.201	0.0280	0.048	0.136	0.0190	0.037
		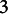	0.636	0.0860	0.094	0.429	0.0650	0.070
			Computation time (min): 42.92			Iterations: 83.83		
		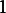	0.428	0.0015	0.021	0.389	0.0012	0.016
	1,500	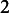	0.192	0.0220	0.044	0.128	0.0175	0.034
		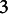	0.451	0.0701	0.073	0.326	0.0520	0.059
			Computation time (min): 25.73			Iterations:80.14		
		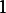	0.389	0.0013	0.017	0.353	0.0010	0.014
DINA	2,000	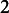	0.144	0.0162	0.033	0.120	0.0130	0.026
		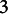	0.300	0.0437	0.051	0.210	0.0345	0.039
			Computation time (min): 62.37			Iterations: 68.76

**Table 6:** Proportion of correctly recovered rows ( 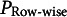 ) and entries ( 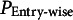 ) for the DINA DeepCDM

*N*	Proportion	Layer 1	Layer 2	Layer 3
1,000	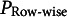	0.709	0.689	0.758
	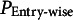	0.957	0.951	0.871
1,500	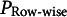	0.852	0.697	0.760
	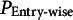	0.990	0.956	0.888
2,000	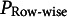	0.878	0.757	0.885
	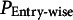	0.993	0.969	0.954

## 5 Real data analysis

To demonstrate the applicability of the exploratory DeepCDM, we analyze student response data from the TIMSS 2019 assessment using a two-layer DeepCDM. Specifically, we examine responses from 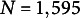 eighth-grade students in the United Arab Emirates who completed Booklet No.1, which includes both mathematics and science items. The dataset comprises responses to 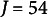 items. Responses were preprocessed into binary indicators of correctness: multiple-choice responses were coded as 1 if correct and 0 otherwise; constructed responses were coded as 1 only if they received the maximum score, and 0 otherwise. According to the *TIMSS 2019 Item Information - Grade 8*, items are classified into two primary domains: mathematics (items 1–28) and science (items 29–54). Each domain further includes four subdomains: mathematics encompasses Number, Algebra, Geometry, and Data & Probability; science includes Biology, Chemistry, Physics, and Earth Science. This hierarchical structure aligns naturally with the two-layer CDM, where the first layer consists of 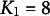 subdomain attributes, and the second layer consists of 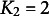 main domain attributes. The item-subdomain-domain assignment structure specified in the TIMSS documentation naturally gives rise to a set of provisional 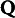 matrices, which are presented in the Supplementary Material. They indeed satisfy the strict identifiability conditions for general DeepCDMs.

To better understand the internal structure of this assessment, we applied a two-layer exploratory DeepCDM to fit the data, using convergence thresholds 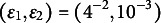 . Given that our primary interest lies in uncovering the hierarchical structure of attributes rather than modeling complex attribute interactions, we selected the main-effect model as our measurement framework. Aligning the number of attributes with 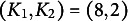 to those specified in the provisional 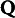 -matrices serves two purposes. First, it allows the exploratory approach to empirically verify the provisional attribute–item mappings, providing evidence for the validity of the test’s original design. Second, the exploratory model maintains sufficient flexibility to identify alternative attribute–item structures, potentially revealing subtle item–attribute relationships not fully anticipated during initial test construction. This dual functionality offers valuable insights for both test validation and future item development.

**Figure 2:**
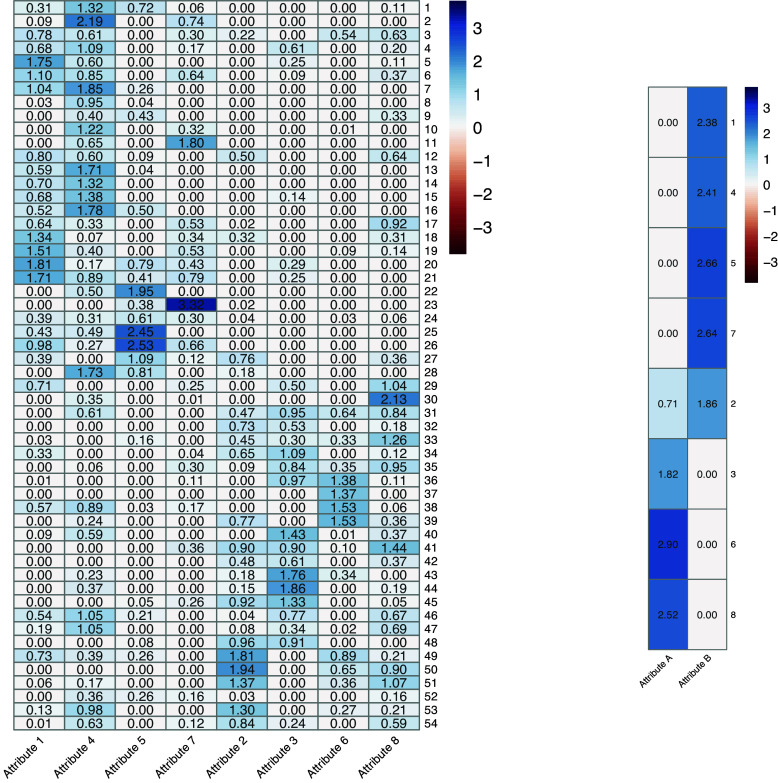
Heatmaps of estimated coefficients from exploratory DeepCDM: First layer (left) and second layer (right).

The attributes in the first layer are numerically labeled from 1 to 8, whereas those in the second layer are labeled as A and B. After estimating the coefficient matrices, we reordered the first-layer attributes to better visualize the underlying block structures. Figure 2 presents heatmaps of the estimated parameters for both layers, from which a distinct structure emerges, aligning closely with the intended test design. Specifically, Figure 2 (left) reveals two clearly defined blocks of non-zero coefficients: the first block associates items 1–28 with Attributes 1, 4, 5, and 7, and the second block links items 29–54 to Attributes 2, 3, 6, and 8. This block structure mirrors TIMSS’s explicit distinction between mathematics and science items. Based on this clear division, we infer that Attributes 1, 4, 5, and 7 represent subdomains belonging to a common domain, while Attributes 2, 3, 6, and 8 form subdomains within another domain. This hierarchical interpretation is further supported by the second-layer heatmap shown in Figure 2 (right), which exhibits sparsity: Attributes 1, 4, 5, and 7 exclusively load onto Meta-Attribute B; Attributes 3, 6, and 8 exclusively load onto Meta-Attribute A; and Attribute 2 uniquely cross-loads onto both Meta-Attributes A and B. This inferred hierarchical structure closely matches the provisional second-layer matrix 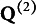 , except for the cross-loading behavior of Attribute 2. Given the item content and structure of 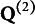 , we conclude that Meta-Attribute A corresponds to the science domain, and Meta-Attribute B to the mathematics domain. The cross-loading of Attribute 2 suggests that this subdomain, despite being associated with science, may also involve mathematical competence during the reasoning process.

Although the exploratory results align closely with the provisional test design, the estimated first-layer attribute structure exhibits some deviations from the provisional 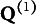 -matrix. Before examining these deviations in detail, we first evaluate the plausibility of our exploratory findings by assessing model fit. Specifically, we compare the exploratory DeepCDM against a confirmatory DeepCDM that directly employs the provisional 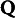 -matrices. These two approaches represent fully data-driven and strictly design-driven modeling, respectively. Consistent with the procedure outlined in our simulation study, model fit is quantified using the BIC. The exploratory DeepCDM achieves a BIC of 89,656, markedly lower than the confirmatory model’s BIC of 94,946. This improvement suggests that exploratory modeling can be beneficial in uncovering item–attribute relationships not fully captured by the original test design.

Next, we investigate the potential item–attribute relationships. Although the secure item content is not publicly available, TIMSS provides detailed metadata for each item, including descriptive labels and topic areas. The item labels offer concise summaries of each item’s content focus, while the topic areas reflect broader curricular domains defined by the TIMSS content framework. These metadata serve as valuable proxies, enabling us to infer the cognitive processes and skills required to answer each item. The complete metadata are provided in the Supplementary Material.

To facilitate interpretation, we focus on the five highest-loading items for each attribute, balancing between representativeness and clarity. Each item is assigned to only one attribute group: specifically, the one for which it has the highest loading value among all attributes, ensuring that item groupings are mutually exclusive and reflect their most salient associations. For each attribute group, we carefully review the content of its assigned items, identify shared cognitive processes, and distill the latent ability the attribute is likely to capture. The distilled attribute names, along with their representative item groups and corresponding cognitive processes, are presented in Table 7.

**Table 7:** Summary of extracted attributes, representative items, and cognitive processes

Index	Extracted attribute	Items	Cognitive process
1	Algebraic Fluency	5, 18, 19, 20, 21	Manipulating algebraic expressions and solving equations in symbolic and applied contexts
2	Scientific Reasoning in Physical Contexts	48, 49, 50, 51, 53	Interpreting experimental conditions and reasoning about physical processes
3	Scientific Classification and Structure Reasoning	34, 40, 43, 44, 45	Categorizing scientific entities based on physical, chemical, or biological properties
4	Applied Quantitative Modeling	2, 7, 13, 16, 28	Applying mathematical concepts to real-world or semi-structured quantitative scenarios
5	Visual Quantitative Reasoning	1, 22, 25, 26, 27	Interpreting quantitative relationships through visual formats, such as graphs, coordinates, and shaded figures
6	Environmental Systems Reasoning	35, 36, 37, 38, 39	Understanding interactions within environmental, planetary, and ecological systems
7	Spatial and Measurement Reasoning	6, 11, 17, 23, 10	Reasoning about shapes, spatial configurations, and geometric measurements
8	Biological and Ecological Reasoning	29, 30, 31, 33, 41	Inferring biological relationships and reasoning through cause–effect processes in ecological systems

To clarify and illustrate our interpretive process, we present two representative examples: Attribute 1 from the mathematics domain and Attribute 2 from the science domain. The detailed interpretation for all eight attributes is provided in the Supplementary Material. Attribute 1 is primarily associated with Items 5, 18, 19, 20, and 21. Based on TIMSS metadata, these items appear to involve tasks, such as expressing the area of a rectangle algebraically, evaluating expressions by substituting values, identifying equivalent algebraic expressions, deriving a formula for stopping distance, and solving for an unknown variable given the perimeter of a triangle. Although these items vary in surface content, they seem to share a common cognitive emphasis on algebraic manipulation and symbolic reasoning. This pattern suggests procedural fluency in algebra, which includes mastering algebraic structures, applying operations accurately, and recognizing equivalent mathematical forms. Accordingly, we interpret Attribute 1 as *Algebraic Fluency*, reflecting the ability to manipulate algebraic expressions and apply fundamental algebraic procedures.

Attribute 2 is primarily associated with Items 48, 49, 50, 51, and 53. According to TIMSS metadata, these items are likely to involve tasks, such as explaining the behavior of gas molecules in an expanding balloon, evaluating appropriate conditions in a heat conduction experiment, reasoning about the effects of planetary gravity on vehicle weight, predicting the behavior of sound in a vacuum, and interpreting evidence related to global warming. While these items span different scientific topics, they appear to share a cognitive focus on reasoning through empirical or hypothetical scenarios, interpreting observations, and evaluating experimental setups. Based on this pattern, we interpret Attribute 2 as *Scientific Reasoning in Physical Contexts*, reflecting systematic reasoning about physical phenomena, empirical data, and conditions relevant to scientific inquiry. As scientific reasoning often draws on mathematical competence, this also supports the observed cross-loading of Attribute 2 onto both science and mathematics domains.

It is important to emphasize that the attribute structure identified through our analysis does not represent the only possible or ideal solution, as the interpretation relies on available metadata rather than direct access to detailed item content. Nevertheless, this empirical analysis illustrates how test data can be examined using an exploratory approach and demonstrates how the resulting attribute structure can be interpreted using accessible metadata. This reflects a common practical scenario, where exploratory results may not fully align with the original test design and detailed item content may be unavailable. By analyzing the derived results through metadata or with expert input, practitioners may uncover findings that offer new perspectives or supplementary insights into the test design.

## 6 Discussion

This article builds a conceptual and methodological bridge between deep generative modeling and cognitive diagnosis. By significantly generalizing the DeepCDMs proposed by Gu (2024) to the challenging exploratory settings, we introduce a new class of models—*exploratory DeepCDMs*—that retain the expressive capacity of DGMs while incorporating the structural constraints, interpretability, and identifiability essential for diagnostic assessment. To enable estimation in this more complex, multi-layer setting with multiple unknown 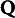 -matrices, we proposed a novel *layer-wise EM algorithm* for regularized maximum likelihood estimation. This algorithm advances the literature by offering a principled, modular framework for learning complex hierarchical latent structures in diagnostic models. Both the algorithm derivation and the identifiability theory of DeepCDMs support a bottom-up, layer-by-layer estimation strategy, making the procedure not only efficient but also theoretically grounded.

A promising direction for future work is to relax the assumption of known latent dimensions across layers. One approach is to incorporate layer-wise dimension selection into the USVT-based initialization, using the largest spectral ratio of singular values, as proposed by Lee & Gu (2025). This procedure can be applied recursively, using estimated or sampled latent attributes from one layer as input to the next, enabling automatic, data-driven dimension selection. Alternative methods, such as the extended BIC (EBIC; Chen & Chen, 2008) and the method of sieves (Shen & Wong, 1994), may also support layer-wise model selection. Another important direction is to determine the number of layers in practice. This may be explored using classical model selection criteria such as BIC. In addition, theoretical guidance may be obtained from the latent structure implied by the model formulation and identifiability conditions. In particular, if the deepest layer is assumed to consist of independent latent variables, one may apply the layer-wise EM procedure upward from the bottom layer and assess whether the estimated latent variables at layer *d* are approximately independent, provided that layer *d* is identifiable. Once a layer is reached at which the estimated latent variables exhibit independence, this layer can be regarded as the deepest layer of the model. This provides a principled, data-driven stopping criterion for determining the appropriate number of layers.

It would also be valuable to extend the model to accommodate polytomous responses and attributes (Chen & de la Torre, 2013; Gao et al., 2021). A similar layer-wise EM algorithm could be developed by applying a one-layer EM procedure for polytomous data at each layer, with corresponding identifiability conditions established for the between-layer 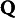 -matrices. Furthermore, continuous latent variables may be introduced at deeper layers to represent broader cognitive abilities. Implementing such an extension would require modifying the current DeepCDM framework to address the computation and identifiability of continuous latent variables, along with the incorporation of continuous inputs for deeper layers. This can be achieved by integrating the methodology of Liu et al. (2025), which proposes a general diagnostic model with higher-order continuous latent structures, along with corresponding identifiability results and an EM-type estimation algorithm. Another useful extension is to develop a stochastic version of the layer-wise EM algorithm. Such variants may offer computational advantages for large-scale, high-dimensional data and serve as a flexible alternative when full E-step computations are costly. More broadly, this work is motivated by the goal of integrating DGMs—and machine learning methods more generally—into cognitive diagnostic modeling. The simulation and empirical results suggest that this integration is a fruitful direction. Moving forward, exploring identifiability in existing DGMs and adapting their algorithms to promote sparsity could benefit not only psychometrics but also other domains where interpretability is crucial.

## Supporting information

Liu and Gu supplementary material Liu and Gu supplementary material

## Supplementary material

The supplementary material for this article can be found at https://doi.org/10.1017/psy.2025.10065.
