# Supplementary material for: Deep Generative Modeling for Cognitive Diagnosis via Exploratory DeepCDMs
Source: Psychometrika. 2025 Dec 17;91(1):151–76. doi: 10.1017/psy.2025.10065 (PMC13121824; doi:10.1017/psy.2025.10065)
Supplement: Liu and Gu supplementary material [file S0033312325100653sup001.pdf]

Supplement to "Deep Generative Modeling for Cognitive Diagnosis via Exploratory DeepCDMs"

Jia Liu and Yuqi Gu

Department of Statistics, Columbia University

This Supplementary Material is organized as follows. Supplement A outlines the identifiability results of DeepCDMs. Supplement C presents the sequences of regularization parameters used in the simulation study. Supplement B presents a comparison of Varimax-based initialization strategies. Supplement D presents additional information for the real data analysis, including the provisional **Q**-matrices, complete metadata and detailed interpretations of all eight extracted attributes presented in Section 5 of the main text.

Supplementary Material

This Supplementary Material is organized as follows. Supplement A outlines the identifiability results of DeepCDMs. Supplement C presents the sequences of regularization parameters used in the simulation study. Supplement B presents a comparison of Varimax-based initialization strategies. Supplement D presents additional information for the real data analysis, including the provisional **Q**-matrices, complete metadata and detailed interpretations of all eight extracted attributes presented in Section 5 of the main text.

A Theoretical Identifiability Conditions

This appendix outlines the identifiability results of DeepCDMs. For the technical proofs of these theoretical results, see Gu (2024).

A.1 Sharp Strict Identifiability Result for DeepDINA

In this subsection, we summarize the sharp necessary and sufficient conditions for the strict identifiability of the DeepDINA model, as established in prior work Gu (2024). The parameter space for the deep-layer population proportions $\boldsymbol{\pi}^{(D)}$ is defined as $\Delta^{2^{K_D}-1} = \left\{\pi^{(D)}_{\boldsymbol{\alpha}_\ell} : \sum_{\ell=1}^{2^{K_D}} \pi^{(D)}_{\boldsymbol{\alpha}_\ell} = 1,\, \pi^{(D)}_{\boldsymbol{\alpha}_\ell} > 0\right\}$. It is assumed that $\pi^{(D)}_{\boldsymbol{\alpha}_\ell} > 0$ for each deep latent profile $\boldsymbol{\alpha}_\ell \in \{0,1\}^{K_D}$—a standard condition consistent with those commonly imposed in single-layer CDMs. We now briefly review the definition of strict identifiability relevant to this setting.

Definition 1 (Strict Identifiability). *An exploratory DeepCDM is said to be strictly identifiable, if the distribution of the observed vector* $\mathbf{R}$ *in* (5) *uniquely determines all of the following: all continuous parameters in the layerwise conditional distributions, the deepest proportion parameters* $\boldsymbol{\pi}^{(D)}$*, and all* $\mathbf{Q}$*-matrices at different depths* $\mathbf{Q}^{(1)}, \dots, \mathbf{Q}^{(D)}$*, up to some column/row permutation.*

A key assumption for DeepDINA's identifiability is the C-R-D conditions. In the traditional DINA model with a saturated attribute framework, these conditions are necessary and sufficient for identifiability, holding in both confirmatory (Gu and Xu, 2019) and exploratory settings (Gu and Xu, 2021). We summarize them below.

(C) **C**ompleteness. A $\mathbf{Q}$-matrix with K columns contains an identity submatrix $\mathbf{I}_K$ after some row permutation. That is, the $\mathbf{Q}$ can be row-permuted to be $\mathbf{Q} = [\mathbf{I}_K, (\mathbf{Q}^*)^\top]^\top$.

(R) **R**epeated-Measurement. Each of the K attributes is measured by at least three items.

(D) **D**istinctness. Assuming Condition (C) holds, after removing the identity submatrix $\mathbf{I}_K$ from $\mathbf{Q}$, the remaining submatrix $\mathbf{Q}^*$ contains K distinct column vectors.

Theorem 1 provides a sharp identifiability result for exploratory DeepDINA with arbitrary depth D, offering the necessary and sufficient conditions on the multiple $\mathbf{Q}$-matrices.

Theorem 1 (DeepDINA). *Consider a ladder-shaped exploratory DeepDINA model with* D *latent layers and* D *between-layer* $\mathbf{Q}$*-matrices* $\mathbf{Q}^{(1)}, \dots, \mathbf{Q}^{(D)}$*. The model is strictly identifiable if and only if each* $\mathbf{Q}^{(d)}$*,* $d = 1, \dots, D$*, satisfies the C-R-D conditions.*

The sharp identifiability conditions in Theorem 1 impose transparent constraints on the $\mathbf{Q}$-matrices, which are also necessary and sufficient for identifying the DeepDINO model due to the duality between DINA and DINO (Chen et al., 2015). These conditions imply that in an identifiable DeepDINA, the layer sizes must satisfy $J > K_1 + \lceil \log_2(K_1) \rceil$ and $K_{d-1} > K_d + \lceil \log_2(K_d) \rceil$ for $d = 2, \ldots, D$ (Gu and Xu, 2021; Gu, 2024). This suggests a progressively shrinking ladder-like sparse architecture for the latent layers as depth increases.

A.2 Strict Identifiability Result for General DeepCDMs

This subsection outlines general strict identifiability conditions for any DeepCDM, including Hybrid DeepCDMs introduced in Section 2.2.

Theorem 2 (General DeepCDM)**.** *Consider an exploratory general DeepCDM with D latent layers and D between-layer $\mathbf{Q}$-matrices $\mathbf{Q}^{(1)}, \ldots, \mathbf{Q}^{(D)}$.* ***Either*** *Condition (S)* ***or*** *Condition (S*) below is sufficient for strict identifiability of the model.*

- *(S) Each $\mathbf{Q}^{(d)}$ can be written as $\mathbf{Q}^{(d)} = [\mathbf{I}_{K_d},\ \mathbf{I}_{K_d},\ \mathbf{I}_{K_d},\ (\mathbf{Q}^{(d)*})^\top]^\top$ after some column/row permutation, where $\mathbf{Q}^{(d)*}$ is an arbitrary $(K_{d-1} - 3K_d) \times K_d$ matrix (potentially empty).*

- *(S*) This condition is the combination of both (S1*) and (S2*) below.*
  - *(S1*) Each $\mathbf{Q}^{(d)}$ can be written as $\mathbf{Q}^{(d)} = [\mathbf{I}_{K_d},\ \mathbf{I}_{K_d},\ (\mathbf{Q}^{(d)*})^\top]^\top$ after some column/row permutation, where $\mathbf{Q}^{(d)*}$ is an arbitrary matrix (potentially empty).*
  - *(S2*) For any two different K_d-dimensional latent patterns $\boldsymbol{\alpha}_c,\ \boldsymbol{\alpha}_\ell \in \{0,1\}^{K_d}$, there exists some $j > 2K_d$ such that $\mathbb{P}(A_j^{(d-1)} = 1 \mid \mathbf{A}^{(d)} = \boldsymbol{\alpha}_c,\ \mathbf{Q}^{(d)},\ \boldsymbol{\theta}^{(d)}) \neq \mathbb{P}(A_j^{(d-1)} = 1 \mid \mathbf{A}^{(d)} = \boldsymbol{\alpha}_\ell,\ \mathbf{Q}^{(d)},\ \boldsymbol{\theta}^{(d)})$, where $\boldsymbol{\theta}^{(d)}$ generically denotes continuous parameters required to fully specify the conditional distribution.*

Theorem 2 is broadly applicable to any DeepCDM, regardless of the diagnostic model used in each layer. Based on the theorem's conditions, the layer sizes must satisfy $J > 2K_1$ and $K_{d-1} > 2K_d$ for $d = 2, \ldots, D$, indicating a progressively shrinking, sparse latent structure as depth increases.

By comparing Theorems 1 and 2, we observe that the sufficient conditions for arbitrary DeepCDMs are stricter than those required for identifying DeepDINA. The next proposition confirms that when a DeepCDM includes a combination of DINA layers and main-effect/all-effect layers, the $\mathbf{Q}$-matrices for the DINA layers only need to satisfy the weaker C-R-D conditions, instead of the stronger Conditions (S) or (S*) in Theorem 2.

Proposition 1 (Hybrid DeepCDM)**.** *Consider a Hybrid DeepCDM with D latent layers and D between-layer $\mathbf{Q}$-matrices $\mathbf{Q}^{(1)}, \ldots, \mathbf{Q}^{(D)}$. If each $\mathbf{Q}^{(d)}$ satisfies the identifiability conditions for the specific diagnostic model that $\mathbf{A}^{(d-1)} \mid \mathbf{A}^{(d)}$ follows (i.e., C-R-D for DINA, (S) or (S*) for main-effect or all-effect model), then the entire DeepCDM is strictly identifiable.*

A.3 Generic Identifiability of Main-effect and All-effect DeepCDMs

Strict identifiability is the strongest notion of identifiability, requiring that parameters be identifiable across the entire parameter space $\mathcal{T}$. A slightly weaker notion, *generic identifiability* (Allman et al., 2009), only requires identifiability almost everywhere in $\mathcal{T}$, allowing non-identifiability on a measure-zero subset $\mathcal{N} \subset \mathcal{T}$. As noted by Allman et al. (2009), generic identifiability is often sufficient for real data analysis and is widely useful in practice. In what follows, we outline the conditions under which *main-effect* and *all-effect* DeepCDMs achieve generic identifiability. We begin by defining *main-effect-based* DeepCDMs.

Definition 2 (Main-effect-based DeepCDMs)**.** *A DeepCDM is said to be "main-effect-based", if the layerwise conditional distribution can be written as:*

$$\mathbb{P}(A_j^{(d-1)} = 1 \mid \mathbf{A}^{(d)} = \boldsymbol{\alpha},\ \mathbf{Q}^{(d)},\ \boldsymbol{\beta}^{(d)}) = f\left(\sum_{k=1}^{K_d} \beta_{j,k}^{(d)} \left\{q_{j,k}^{(d)} \alpha_k\right\} + \cdots\right).$$

where $f(\cdot)$ is a link function, and the " $\cdots$ " refers to potentially more terms such as the interaction-effects of the α_k's and the intercept.

Note that Main-effect-based DeepCDMs also covers All-effect DeepCDMs, because the latter also incorporate the main effects of attributes. DeepDINA is not a main-effect-based

DeepCDM since it lacks the main-effect coefficients, like $\beta_{j,k}^{(d)}$, outlined in Definition 2. These coefficients are key to achieving generic identifiability and allow relaxing the condition that each $\mathbf{Q}^{(d)}$ must contain a submatrix $\mathbf{I}_{K_d}$ (Gu and Xu, 2020; Chen et al., 2020). Next, we formally define and establish the generic identifiability of main-effect-based DeepCDMs.

Definition 3. *Define the allowable constrained parameter space for $\boldsymbol{\beta}^{(d)}$ in Definition 2 under the binary matrix $\mathbf{Q}^{(d)}$ as*

$$\Omega_{\text{main}}(\boldsymbol{\beta}^{(d)};\, \mathbf{Q}^{(d)}) = \{\beta_{j,k}^{(d)} \neq 0 \text{ if } q_{j,k}^{(d)} = 1;\ \text{and } \beta_{j,k}^{(d)} = 0 \text{ if } q_{j,k}^{(d)} = 0\}. \tag{1}$$

The continuous parameters and the $\mathbf{Q}$-matrices are said to be generically identifiable if the set of unidentifiable continuous parameters has measure zero with respect to the Lebesgue measure on their parameter space $\cup_{d=1}^{D}\Omega_{\text{main}}(\boldsymbol{\beta}^{(d)};\, \mathbf{Q}^{(d)}) \cup \Delta^{2^{K_D}-1}$.

Theorem 3. *Consider a main-effect-based DeepCDM. Suppose each $\mathbf{Q}^{(d)}$ can be written as $\mathbf{Q}^{(d)} = [(\mathbf{Q}_1^{(d)})^\top, (\mathbf{Q}_2^{(d)})^\top, (\mathbf{Q}^{(d)*})^\top]^\top$ after some column/row permutation and satisfies the following conditions. Then the main-effect-based DeepCDM is generically identifiable.*

(G1) Each $\mathbf{Q}_m^{(d)}$ $(m = 1, 2)$ has size $K_d \times K_d$ and takes the following form:

$$\mathbf{Q}_m^{(d)} = \begin{pmatrix} 1 & * & \cdots & * \\ * & 1 & \cdots & * \\ \vdots & \vdots & \ddots & \vdots \\ * & * & \cdots & 1 \end{pmatrix}, \quad m = 1, 2; \quad d = 1, \ldots, D.$$

That is, $\mathbf{Q}_1^{(d)}$ and $\mathbf{Q}_2^{(d)}$ each has all the diagonal entries equal to one, whereas any off-diagonal entry is free to be either one or zero.

(G2) The $(K_{d-1} - 2K_d) \times K_d$ submatrix $\mathbf{Q}^{(d)*}$ in $\mathbf{Q}^{(d)}$, $d = 1, \ldots, D$, satisfies that each column contains at least one entry of "1".

Theorem 3 relaxes the strict identifiability conditions from Theorem 2 by removing the requirement for any $\mathbf{Q}^{(d)}$ to contain an identity submatrix $\mathbf{I}_{K_d}$. Moreover, these generic

identifiability conditions suggest a shrinking latent structure as depth increases, since (G1) and (G2) imply that $J > 2K_1$ and $K_d > 2K_{d+1}$ for $d = 1, \dots, D-1$.

B Comparison of Varimax-based Initialization Strategies

To compare the performance of the initialization procedures described in Section 3.3, which differ in how the Varimax rotation is applied, we conducted a simulation under the main-effect CDM setting to evaluate the performance of the two strategies in parameter estimation and **Q**-matrix recovery. We refer to our proposed method, which applies Varimax to the right singular matrix, as the *Right–Varimax Initialization (Ini–RV)*, and the alternative method, which applies Varimax directly to the scaled singular-value–weighted matrix, as the *Direct–Varimax Initialization (Ini–DV)*. To avoid randomness introduced by layer-wise EM updates in multi-layer DeepCDMs, we focus on a single-layer case that shares the same configuration as the bottom layer in the main simulation section. For Ini–DV, we follow the procedure outlined in Zhang et al. (2020) to obtain $\frac{1}{\sqrt{N}}(\widehat{\tau}_1\widehat{v}_1, \dots, \widehat{\tau}_{K_{d-1}}\widehat{v}_{K_{d-1}})$. We then apply the Varimax rotation to this matrix, followed by thresholding and sign-flip adjustment using the same strategy as in Step 9 of Algorithm 2.

We report the RMSE and aBias of $\boldsymbol{\beta}^{(1)}$ to assess parameter estimation accuracy, and the proportion of correctly recovered entries in $\mathbf{Q}^{(1)}$ to assess **Q**-matrix recovery. As shown in Table B.1, Ini–RV achieves smaller RMSE and aBias than Ini–DV, indicating higher estimation accuracy. Moreover, Ini–RV demonstrates substantially better **Q**-matrix recovery performance. For further examination, we present heatmaps of the initialized coefficient matrices after applying the Hungarian permutation in a representative replication. As illustrated in Figure B.1, many small but nonzero values remain unthresholded in Ini–DV, contributing to its poorer performance in parameter estimation and **Q**-matrix recovery. Meanwhile, for entries with nonzero true values, Ini–DV estimates tend to be closer to the true magnitudes, whereas Ini–RV estimates are slightly smaller. Nevertheless, both methods yield $\boldsymbol{\beta}^{(1)}$ with a

sparse pattern, and the estimated values are reasonably close to the true ones.

Method	Metric	$N = 1000$	$N = 1500$	$N = 2000$
Ini–RV	$RMSE_{\boldsymbol{\beta}^{(1)}}$	0.469	0.465	0.459
	$ABias_{\boldsymbol{\beta}^{(1)}}$	0.173	0.170	0.167
	$P_{\mathbf{Q}}$	0.822	0.823	0.825
Ini–DV	$RMSE_{\boldsymbol{\beta}^{(1)}}$	0.517	0.498	0.500
	$ABias_{\boldsymbol{\beta}^{(1)}}$	0.385	0.365	0.360
	$P_{\mathbf{Q}}$	0.249	0.263	0.264

Table B.1: Comparison of Right–Varimax (Ini–RV) and Direct–Varimax (Ini–DV) initializations under the main-effect CDM for different sample sizes. Reported are RMSE and aBias of $\boldsymbol{\beta}^{(1)}$ and the proportion of correctly recovered $\mathbf{Q}^{(1)}$ entries ($P_{\mathbf{Q}}$).

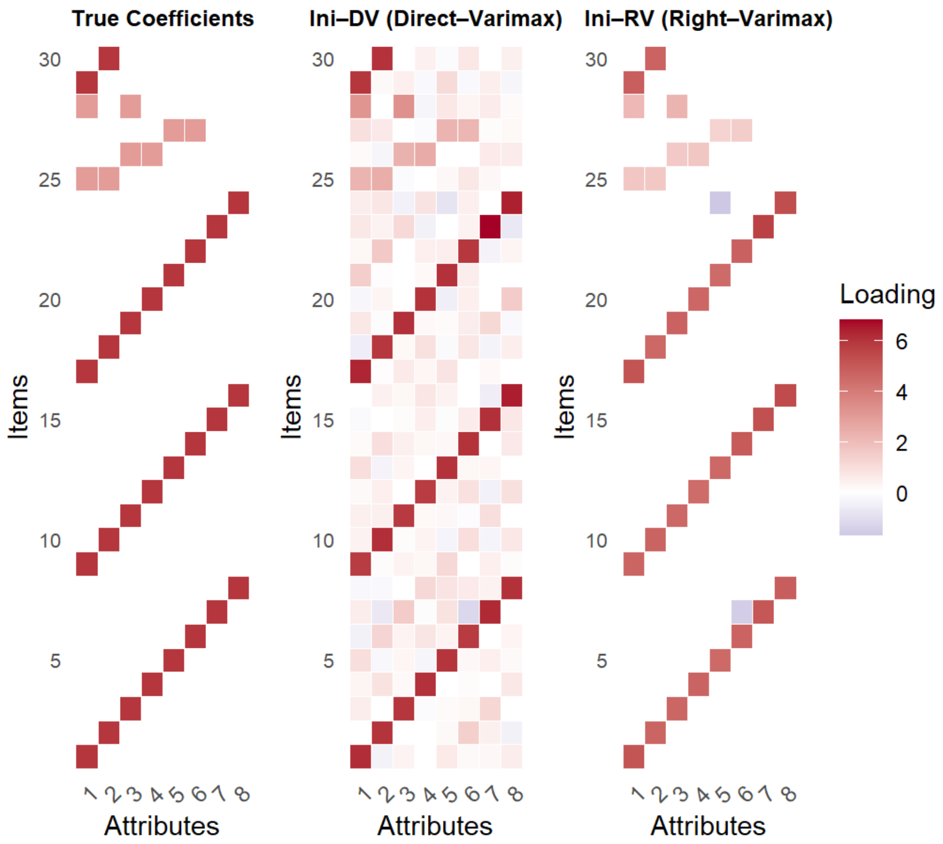

Figure B.1: Heatmaps of the true coefficient matrix $\boldsymbol{\beta}^{(1)}$ and its initializations: (a) True coefficients, (b) Direct–Varimax (Ini–DV), and (c) Right–Varimax (Ini–RV). Sample size: $N = 1000$.

C Sequences of Regularization Parameters Used in the Simulation Study

Table C.1 presents the sequences of scaled regularization parameters $N \cdot s_d$ used in the simulation study across different sample sizes and layers. In this implementation, the regularization sequence was selected through a data-driven grid search guided by the Bayesian Information Criterion (BIC). The candidate grid was determined based on preliminary simulation experiments to identify a reasonable value range for each setting. For each simulation condition, a moderate grid centered around these empirically derived values was constructed, and the final sequence corresponded to those yielding the smallest BIC values. These sequences were applied consistently across all three measurement model settings—main-effect, all-effect, and DINA—to ensure comparability and avoid manual adjustment. We acknowledge that, as the choice of sequence depends on the grid's center and granularity and may vary across model settings, the resulting sequence is satisfactory but not necessarily optimal.

Sample size	Layer		
	1	2	3
1000	$(0.010, 0.011, 0.012)$	$(0.010, 0.011, 0.012)$	$(0.015, 0.016, 0.017)$
1500	$(0.009, 0.010, 0.011)$	$(0.009, 0.010, 0.011)$	$(0.014, 0.015, 0.016)$
2000	$(0.008, 0.009, 0.010)$	$(0.008, 0.009, 0.010)$	$(0.013, 0.014, 0.015)$

Table C.1: Sequences of scaled regularization parameters $N \cdot s_d$ used in the simulation study, across different sample sizes and layers.

D Supplement for Data Analysis

In this appendix, we provide supplementary information for the real data analysis in Section 5. Tables D.1 and D.2 present the provisional $\mathbf{Q}$-matrices derived from the TIMSS assessment design. Table D.3 lists metadata for each item, including descriptive labels and associated topic areas. In addition, detailed interpretations of the eight extracted attributes in Table 7 of the main text are provided below.

Item ID	Number	Algebra	Geometry	Data & Prob.	Biology	Chemistry	Physics	Earth Science
1	1	0	0	0	0	0	0	0
2	1	0	0	0	0	0	0	0
3	1	0	0	0	0	0	0	0
4	1	0	0	0	0	0	0	0
5	0	1	0	0	0	0	0	0
6	0	1	0	0	0	0	0	0
7	0	1	0	0	0	0	0	0
8	0	1	0	0	0	0	0	0
9	0	0	1	0	0	0	0	0
10	0	0	1	0	0	0	0	0
11	0	0	1	0	0	0	0	0
12	0	0	0	1	0	0	0	0
13	0	0	0	1	0	0	0	0
14	1	0	0	0	0	0	0	0
15	1	0	0	0	0	0	0	0
16	1	0	0	0	0	0	0	0
17	1	0	0	0	0	0	0	0
18	0	1	0	0	0	0	0	0
19	0	1	0	0	0	0	0	0
20	0	1	0	0	0	0	0	0
21	0	1	0	0	0	0	0	0
22	0	1	0	0	0	0	0	0
23	0	0	1	0	0	0	0	0
24	0	0	1	0	0	0	0	0
25	0	0	1	0	0	0	0	0
26	0	0	0	1	0	0	0	0
27	0	0	0	1	0	0	0	0
28	0	0	0	1	0	0	0	0
29	0	0	0	0	1	0	0	0
30	0	0	0	0	1	0	0	0
31	0	0	0	0	1	0	0	0
32	0	0	0	0	1	0	0	0
33	0	0	0	0	0	1	0	0
34	0	0	0	0	0	0	1	0
35	0	0	0	0	0	0	1	0
36	0	0	0	0	0	0	1	0
37	0	0	0	0	0	0	0	1
38	0	0	0	0	0	0	0	1
39	0	0	0	0	1	0	0	0
40	0	0	0	0	1	0	0	0
41	0	0	0	0	1	0	0	0
42	0	0	0	0	1	0	0	0
43	0	0	0	0	1	0	0	0
44	0	0	0	0	0	1	0	0
45	0	0	0	0	0	1	0	0
46	0	0	0	0	0	1	0	0
47	0	0	0	0	0	1	0	0
48	0	0	0	0	0	0	1	0
49	0	0	0	0	0	0	1	0
50	0	0	0	0	0	0	1	0
51	0	0	0	0	0	0	1	0
52	0	0	0	0	0	0	0	1
53	0	0	0	0	0	0	0	1
54	0	0	0	0	0	0	0	1

Table D.1: First-layer provisional $\mathbf{Q}$-matrix $\mathbf{Q}^{(1)}_{54\times 8}$ for item booklet No.1 in TIMSS 2019 eighth grade assessment.

Subdomains \ Main Domains	Mathematics	Science
Number	1	0
Algebra	1	0
Geometry	1	0
Data and Probability	1	0
Biology	0	1
Chemistry	0	1
Physics	0	1
Earth Science	0	1

Table D.2: Second-layer $\mathbf{Q}$-matrix $\mathbf{Q}^{(2)}_{8\times 2}$ for TIMSS 2019 eighth grade assessment.

Item ID	Topic Area	Label
1	Fractions and Decimals	Octagon with equivalent shading
2	Integers	Time when Pat finishes last lap; Percentage of laps finished
3	Integers	Multiples of 3
4	Fractions and Decimals	Convert decimal to a fraction
5	Expressions, Operations, and Equations	Expression for area of rectangle
6	Expressions, Operations, and Equations	Expression with exponents of y
7	Relationships and Functions	Number of matches for figure 10; Rule for number of matches
8	Relationships and Functions	Graph of y = 2x
9	Geometric Shapes and Measurements	Rotation and reflection
10	Geometric Shapes and Measurements	Surface area of the prism
11	Geometric Shapes and Measurements	Value of angle x outside triangle
12	Probability	Number of balls in a bag
13	Data	Liv's smartphone use; Smartphone use listening to music
14	Integers	Statements for all values of integer a (DERIVED)
15	Fractions and Decimals	Arrow to show 5/12 on number line
16	Fractions and Decimals	Value of fraction X in square
17	Ratio, Proportion, and Percent	Number of blue beads on bracelet
18	Expressions, Operations, and Equations	Value of 2(6x - 3y)
19	Expressions, Operations, and Equations	Expression equivalent to 2y + 6xy2
20	Expressions, Operations, and Equations	Formula for stopping distance
21	Expressions, Operations, and Equations	Value of x given perimeter of triangle ABC
22	Relationships and Functions	Additional point on a straight line
23	Geometric Shapes and Measurements	Value of angle x in a quadrilateral
24	Geometric Shapes and Measurements	Methods of folding paper- height, diameter, surface area
25	Geometric Shapes and Measurements	Coordinates to complete KLMN- x coordinate, - y coordinate
26	Data	Mean temperature for 5 days
27	Data	Best graph for town information - jobs, boys and girls, population
28	Data	Bar graph of newspaper sales
29	Cells and Their Functions	Organism with cell walls
30	Ecosystems	How decomposers get energy
31	Ecosystems	Organism that competes with humans
32	Ecosystems	Garden with bird feeder: cat+birds, cat+birds, cat+mouse
33	Properties of Matter	Why Solution 2 is paler than 1

Item ID	Topic Area	Label
34	Physical States and Changes in Matter	Which is a physical change
35	Electricity and Magnetism	Model flashlight: Bulb won't light; 2 parallel bulbs; Comparison
36	Electricity and Magnetism	Two bar magnets repelling
37	Earth in the Solar System and the Universe	Planets: Shortest day length; Distance from Sun
38	Earth's Structure and Physical Features	Temperature outside an airplane
39	Ecosystems	Relationship between insects and flowering plants
40	Cells and Their Functions	Where in a cell DNA replication occurs
41	Ecosystems	Increase green space as carbon dioxide increases
42	Ecosystems	Why leaves' masses decreased
43	Characteristics and Life Processes of Organisms	Classify animals based on a single characteristic, Identify the characteristic used to classify animals
44	Composition of Matter	Location of subatomic particles
45	Composition of Matter	Order elements from smallest to largest atomic num
46	Properties of Matter	Acidic, basic, or neutral solution
47	Properties of Matter	Mixing an acid and base solution
48	Physical States and Changes in Matter	Gas molecules in an expanding balloon
49	Energy Transformation and Transfer	Things Tom should do (DERIVED): same type of wax on both rods, higher flame for the copper rod, paperclips from different materials, etc.
50	Motion and Forces	Vehicle with different weights on different planets
51	Light and Sound	Cell phone in a vacuum
52	Earth's Structure and Physical Features	Why balloon gets bigger as it rises
53	Earth's Processes, Cycles, and History	Evidence of global warming
54	Earth's Processes, Cycles, and History	Natural resource formation shown in diagrams

Table D.3: Metadata for TIMSS Items in Booklet 1

Attribute 1 is primarily associated with Items 5, 18, 19, 20, and 21. Based on TIMSS metadata, these items appear to involve tasks such as expressing the area of a rectangle algebraically, evaluating expressions by substituting values, identifying equivalent algebraic expressions, deriving a formula for stopping distance, and solving for an unknown variable given the perimeter of a triangle. Although these items vary in content, they share a common cognitive emphasis on algebraic manipulation and symbolic reasoning. This pattern suggests procedural fluency in algebra, which includes mastering algebraic structures, applying oper-

ations accurately, and recognizing equivalent mathematical forms. Accordingly, we interpret Attribute 1 as *Algebraic Fluency*, reflecting the ability to manipulate algebraic expressions and apply fundamental algebraic procedures.

Attribute 2 is primarily associated with Items 48, 49, 50, 51, and 53. According to TIMSS metadata, these items are likely to involve tasks such as explaining the behavior of gas molecules in an expanding balloon, evaluating appropriate conditions in a heat conduction experiment, reasoning about the effects of planetary gravity on vehicle weight, predicting the behavior of sound in a vacuum, and interpreting evidence related to global warming. While these items span different scientific topics, they share a cognitive focus on reasoning through empirical or hypothetical scenarios, interpreting observations, and evaluating experimental setups. Based on this pattern, we interpret Attribute 2 as *Scientific Reasoning in Physical Contexts*, reflecting systematic reasoning about physical phenomena, empirical data, and conditions relevant to scientific inquiry.

Attribute 3 corresponds to Items 34, 40, 43, 44, and 45, which, based on their metadata, appear to involve identifying physical changes, locating cellular processes, classifying organisms, recognizing subatomic structures, and ordering elements by atomic number. These items seem to require categorization and structural understanding of scientific entities across biology, chemistry, and physics. The common cognitive emphasis lies in classification and the organization of scientific knowledge. Accordingly, we interpret Attribute 3 as *Scientific Classification and Structure Reasoning*, reflecting the ability to sort and organize domain-specific information using scientific criteria.

Attribute 4 includes Items 2, 7, 13, 16, and 28. These items are likely to involve applying mathematical reasoning to contextualized or real-world situations, such as interpreting percentages and time, identifying numerical patterns, analyzing device usage, working with fractions, and interpreting a bar graph of newspaper sales. The shared emphasis appears to be on translating semi-structured scenarios into quantitative representations. We therefore interpret Attribute 4 as *Applied Quantitative Modeling*, referring to the ability to construct and use mathematical representations to understand and reason about contextualized quan-

titative information.

Attribute 5 is defined by Items 1, 22, 25, 26, and 27. According to metadata, these items appear to involve reasoning with shaded figures, identifying linear patterns, completing coordinate shapes, computing averages, and selecting appropriate graphs. The shared cognitive emphasis is on interpreting visual or spatial representations to extract quantitative meaning. As such, we interpret Attribute 5 as *Visual Quantitative Reasoning*, which highlights the ability to engage in quantitative thinking through visual cues and data structures.

Attribute 6 consists of Items 35, 36, 37, 38, and 39. These items are associated with topics such as electrical circuits, magnetic forces, planetary properties, atmospheric conditions, and ecological interactions. While varying in scientific content, they collectively seem to require reasoning about the dynamic relationships that govern natural or environmental systems. Therefore, we interpret Attribute 6 as *Environmental Systems Reasoning*, reflecting the process of analyzing complex physical and ecological interactions.

Attribute 7 corresponds to Items 6, 11, 17, 23, and 10. Based on their descriptions, these items likely require reasoning about spatial configurations, angle relationships, proportional reasoning, and surface area computation. The common cognitive demand appears to be spatial visualization integrated with quantitative reasoning. We interpret Attribute 7 as *Spatial and Measurement Reasoning*, denoting the ability to reason about shape, measurement, and geometric relationships. We note that Item 6, while involving algebraic expressions with exponents, may not directly reflect spatial or measurement reasoning. Its inclusion in this group may reflect empirical overlap rather than conceptual alignment and should therefore be interpreted with caution.

Attribute 8 is associated with Items 29, 30, 31, 33, and 41. These items involve topics such as cellular structure, energy flow in ecosystems, species interactions, substance concentration, and environmental impact. While the specific topics vary, they seem to require reasoning about biological mechanisms and ecological cause-effect patterns. Thus, we interpret Attribute 8 as *Biological and Ecological Reasoning*, reflecting the ability to understand and infer relationships and processes within living systems.

References

Allman, E. S., Matias, C., and Rhodes, J. A. (2009). Identifiability of parameters in latent structure models with many observed variables. *The Annals of Statistics*, 37(6A):3099–3132.

Chen, Y., Culpepper, S. A., and Liang, F. (2020). A sparse latent class model for cognitive diagnosis. *Psychometrika*, 85(1):121–153.

Chen, Y., Liu, J., Xu, G., and Ying, Z. (2015). Statistical analysis of Q-matrix based diagnostic classification models. *Journal of the American Statistical Association*, 110(510):850–866.

Gu, Y. (2024). Going deep in diagnostic modeling: Deep cognitive diagnostic models (deepcdms). *Psychometrika*, 89(1):118–150.

Gu, Y. and Xu, G. (2019). The sufficient and necessary condition for the identifiability and estimability of the DINA model. *Psychometrika*, 84(2):468–483.

Gu, Y. and Xu, G. (2020). Partial identifiability of restricted latent class models. *Annals of Statistics*, 48(4):2082–2107.

Gu, Y. and Xu, G. (2021). Sufficient and necessary conditions for the identifiability of the Q-matrix. *Statistica Sinica*, 31:449–472.

Zhang, H., Chen, Y., and Li, X. (2020). A note on exploratory item factor analysis by singular value decomposition. *Psychometrika*, 85(2):358–372.
